# Decorating probiotics with a triggerable and catalytic shell for synergistically enhanced colitis biotherapy

**DOI:** 10.1016/j.mtbio.2025.101861

**Published:** 2025-05-12

**Authors:** Zhishu Li, Xinlin Wei, Wenting Chen, Xuelian Qiu, Jieyan Shi, Yu Li, Zhixuan Wang, Xiaolin Chen, Yuepeng Wang, Lizeng Cheng, Bo Teng, Harold Corke, Bo-Bo Zhang, Qiongqiong Yang

**Affiliations:** a Department of Biology, College of Science, Shantou University, Shantou, 515063, Guangdong, China; b Guangdong Yichao Biological Co., Ltd., Shantou, 515064, Guangdong, China; c Guangdong Provincial Key Laboratory of Marine Biotechnology, Institute of Marine Sciences, Shantou University, Shantou, 515063, China; d Shantou Key Laboratory of Marine Microbial Resources and Interactions with Environment, Shantou University, Shantou, 515063, China; e Guangdong Branch of State Key Laboratory for Esophageal Cancer Prevention and Treatment, Shantou, 515063, Guangdong, China; f School of Agriculture and Biology, Shanghai Jiao Tong University, Shanghai, 200240, China; g Biotechnology and Food Engineering Program, Guangdong Technion-Israel Institute of Technology, Shantou, 515063, Guangdong, China; h Faculty of Biotechnology and Food Engineering, Technion-Israel Institute of Technology, Haifa, 320003, Israel

**Keywords:** Probiotic, Gut microbiota, Ulcerative colitis, On-demand reactivation, Reactive oxygen species

## Abstract

Probiotics have creditableness in adjunctive therapy for ulcerative colitis, but their physiological activity and alleviation efficiency are frequently compromised by gastrointestinal conditions and oxidative stress microenvironments. Here, we developed a probiotic-based therapeutic that synergistically ameliorates colonic structure, intestinal redox, and microbiota homeostasis. Probiotics were initially coated with a metal-phenolic shell to provide a network for spatially loading antioxidant enzymes, and then wrapped with a pH-triggerable enteric layer to enable on-demand reactivation in the inflamed colon. Upon oral administration, the pH-triggerable enteric layer efficiently enhanced gastric acid resistance and on-demand release of enzyme-loaded probiotics in the inflamed colon, enabling efficient reactive oxygen species scavenging and salutary gut microbiota regulation at the lesional regions. In multiple mouse models, this therapeutic normalized immunologic balance and mitigated colonic damage with high efficacy, thus representing a powerful strategy for colitis therapy.

## Graphical abstract

**Figure:**
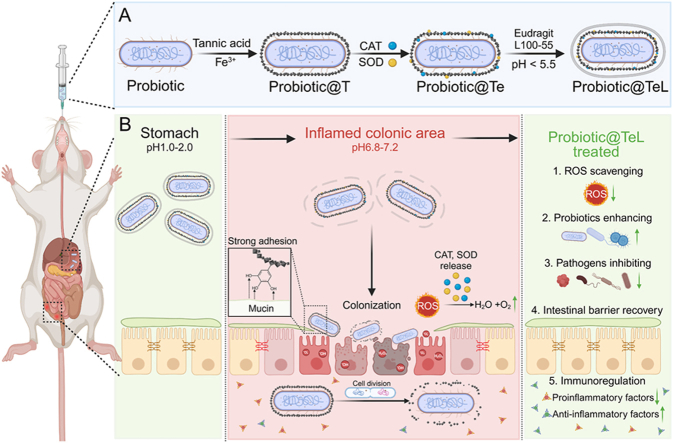

## 1 Introduction

Ulcerative colitis (UC), classified with Crohn's disease (CD) as an inflammatory bowel disease (IBD), is a chronic and recurrent inflammatory disorder affecting gastrointestinal (GI) tract [1]. Over the past few decades, these debilitating disorders have increasingly affected adolescents, with a rising incidence and prevalence globally, placing a huge healthcare burden on countries and societies [2,3]. Patients are advised to adapt a step-up strategy using chemical drugs or monoclonal antibodies to reach the desired remission, as there is currently no clinically available or effective cure for UC [4,5]. However, owing to the complexity of the GI structure and the impediment of mucus, present small-molecule drugs and bio-pharmaceuticals have notable limitations in ameliorating intestinal inflammation [6]. The prolonged and frequent use of these drugs may incur serious side effects, such as nausea, headaches, opportunistic infections, autoimmunity, and liver toxicity [[7], [8], [9]]. Therefore, there is a pressing need to design and develop novel and low-burden remedial strategies for UC, which is characterized by dysregulated gut microbiota, excessive production of ROS, and impaired colonic epithelium [[10], [11], [12]]. The strategy that simultaneously addresses the three hallmarks of UC while causing minimal systemic side effects represents a promising approach to the therapeutic challenges posed by UC.

Bacteria therapy, especially probiotic therapy, is a promising treatment option for patients with GI disorders [[13], [14], [15], [16]]. Several clinical trials have demonstrated the successful and safe delivery of probiotics to the target area of colitis, leading to improved functional outcomes. This effectiveness is attributed to their living characteristics, including colonization, rapid proliferation, positive regulation of the composition and function of microbiota, and modulation of host immunity and metabolism [17]. However, the therapeutic efficacy of probiotics leaves much to be desired, due to several unavoidable factors during oral administration of probiotics, such as competition for colonization, environmental assaults (pH, lytic enzymes, ultraviolet irradiation, and antibiotics), and non-adhesive host bio-interfaces [18,19]. To overcome the limitations, single-cell nanoencapsulation (SCNE) methods have been applied to therapies aimed at targeted delivery of probiotics to inflamed colons, particularly addressing the microenvironment stresses [[20], [21], [22], [23]].

Among the materials and processes available for SCNE and other bioencapsulation techniques (*e.g.*, layer-by-layer assembly [24] and bioinspired silicification [25]), metal-organic frameworks (MOFs) have recently captured considerable interest following Falcaro's pioneering work [26,27]. When it comes to interfacing with bioentities, the MOFs possess distinct and beneficial properties, including compositional and topological tunability, porosity, and the ability to be functionalized, all complemented by their high biocompatibility [28]. Recent advancements in SCNE focus on developing methods to arm living cells with exogenous catalytic functions, endowing them with characteristics of proactively communicating with and manipulating the fickle surroundings *in vivo* and *in vitro* [29,30]. For example, polydopamine, laccase, and tannic acid (TA) were employed by Huang et al. in encapsulating *Chlorella pyrenoidosa* through successive deposition [31]. In their system, the O_2_ consumption by laccase during the oxidation of TA reprogrammed microbial activities, shifting them from O_2_ production *via* photosystem Ⅱ to anaerobic H_2_ production. Inspired by the excellent MOF properties and their potential to be modified to catalytic shells, we hypothesize that embedding antioxidant enzymes within MOF shells, combined with a pH-responsive material, can effectively address the specific characteristics of UC, such as high levels of ROS, pH 5–7, and cationic colonic mucus of the patchy inflammatory region, thereby providing a productive treatment for UC.

In this study, we established a platform capable of on-demand reactivation of probiotics in the inflamed colon while simultaneously eliminating excessive ROS and regulating gut microbiota homeostasis. Probiotics *Escherichia coli* Nissle 1917 GDMCC 1.3181 (EcN) and *Lactobacillus paracasei* CGMCC 1.2744 (LP) were sequentially mixed with Fe^3+^ and TA, creating a film on their surface (probiotic@T). The remarkable adhesive peculiarity of TA provided by its pyrogallol and catechol groups can augment the colonization capacity of probiotics in the intestine [32,33]. Subsequently, catalase (CAT) and superoxide dismutase (SOD) were embedded into the straticulate shell (probiotic@Te), empowered shell with capabilities of ROS scavenging and inflammation alleviation. Benefited from the protective effect of MOF scaffolds, the embedded enzymes can resist to various stress environments that are detrimental to deliberate native enzymes, even at protein-denaturing conditions [34,35]. To realize the accurate delivery of oral probiotics to the colon for reinforcing bacteriotherapy, we further encapsulated probiotic@Te with Eudragit L100-55 (probiotic@TeL), which is a clinically used enteric coating that precipitates in acidic environments and dissolves in the intestine (pH > 6) [36,37]. This coating protects probiotics from acid attacks in the stomach and enables the targeted release of probiotics in the intestine. Our strategy endows probiotics with triggerable and catalytic nanoshells, which can separate probiotics from the hash gastric environment and trigger the release of EcN@Te upon delivery to the intestine; then the CAT and SOD embedded within the shells can scavenge excess ROS and simultaneously release probiotics with negligible damage. Dissociated polyphenols can be decomposed and metabolized by microbiota into more active metabolites, representing prebiotic-like characteristics [38,39]. The therapeutic effect on colitis is further enhanced by the synergistic effect of catalytic therapy, intestinal immunomodulation, and microbiota remodeling. Our research demonstrated that EcN@TeL could resist the hostile environment in the digestive tract and target the formation of intestinal ecological niche, effectively preventing and alleviating DSS-induced UC symptoms including weight loss, disease activity index (DAI) elevating, colon shortening, ROS overproduction, and restoring the intestinal barrier. Furthermore, the dysbiosis of intestinal flora and the short-chain fatty acids (SCFAs) were rebuilt in the treated mice. This strategy is applicable to various strains and suitable for implantation with diverse enzymes. We foresee that encapsulating probiotics with responsive and catalytic shells will serve as a versatile method for creating biologically functional probiotics that offer improved bioavailability and enhanced treatment efficacy. We believe that these shell-coated probiotics represent a distinctive tool for various biomedical applications.

## 2 Materials and methods

### 2.1 Reagents

Tannic acid, Iron (III) chloride hexahydrate (FeCl_3_ • 6H_2_O) were purchased from Sigma (USA). Hoechst 33342 was bought from Beyotime (Shanghai, China). Live or Dead Cell Viability Assays were bought from AAT Bioquest (USA). Pepsin, Trypsin, 3-Morpholinopropanesulfonic Acid (MOPS), CAT, SOD, and Tween-80 were purchased from Macklin (Shanghai, China). Phosphate-buffered saline (PBS) was bought from Procell (Wuhan, China). Calcium chloride, Glucose, Sodium acetate trihydrate, Bitter salt, and Manganese sulfate monohydrate were purchased from Xilong Scientific (Shantou, China). Eudragit L100-55 (L100) was bought from Evonik (Germany). Cy5.5-labeled bovine serum albumin was purchased from Xi'an Qiyue Biological Technology Co., Ltd. Fluorescence probe 1,1-dioctadecyl-3,3,3,3-tetramethylindotricarbocyaine iodide (DIR) was purchased from MCE (Shanghai, China). Tryptone, Yeast extract and Casitone were bought from Angelyeast (Yichang, China). Technical agar and Beef extract powder were purchased from HuanKai Microbio (Guangdong, China). Diammonium hydrogen citrate, Dipotassium hydrogen phosphate trihydrate and KH_2_PO_4_ were bought from GHTECH (Guangdong, China). Fluorescein Isothiocyanate (FITC) and Dextran Sulfate Sodium Salt (DSS, MW:36000–50000) were bought from YEASEN (Shanghai, China). Anti-MUC2 Rabbit pAb, Anti-Myeloperoxidase Rabbit pAb, Anti-Occludin Rabbit pAb, Anti-ZO1 tight junction protein Rabbit pAb and HRP conjugated Goat Anti-Rabbit IgG (H + L) were purchased from Servicebio (Wuhan, China). Primers were bought from Sangon (Shanghai, China).

### 2.2 Strain and plasmid

*Escherichia coli* Nissle 1917 (EcN, GDMCC 1.3181) was purchased from Guangdong Microbial Culture Collection Center (Guangdong, China), *Lactobacillus paracasei* (LP, CGMCC 1.2744) was bought from China General Microbiological Culture Collection Center (Beijing, China). EcN were cultured on LB solid plates with 1.5 % agar, while LP were cultured on MRS solid plates with 1.3 % agar. Probiotics were added to LB or MRS liquid medium and proliferated overnight at 37 °C with shaking rate of 150 rpm before each experiment. Plasmid pUC19-EGFP was provided by domestic suppliers and used as received.

### 2.3 Preparation of EcN@TeL

Overnight cultured EcN cells were collected by centrifugation at 8000 rpm for 4 min and washed with PBS twice. Bacterial solution (250 μL) was mixed with FeCl_3_ (5 mM, 125 μL) and TA solution (5 mM, 125 μL), and then vibrated for 15 min to form a stable shell on the bacterial surface, the EcN@T suspension was centrifuged and washed with PBS twice. Afterward, 210 U CAT and 100 U SOD were added to the suspension and incubated for 10 min with shaking. After being washed by PBS and centrifuged, EcN@Te was resuspended with 900 μL of ice-cold calcium phosphate suspension (12.5 mM) and 500 μL of L100 (1 mg/mL), with 5 min vortex after each addition, 0.1 M HCl was added to the system to adjust pH 5.5 to form a protective L100 layer on the surface of EcN@Te. Then EcN@TeL was collected by centrifugation, washed, and resuspended by double distilled water (ddH_2_O) with pH < 5 for the following experiments.

### 2.4 Artificial degradation of TA and L100 shells

The degradation of L100 was triggered by the double incubation of EcN@TL in PBS with shaking at 150 rpm for 10 min. EcN@T was resuspended in 1 mL ascorbic acid (AA, 10 mM) and shaken for 10 min. Ascorbic acid can compete with Fe^3+^-TA for iron ions and dissociate it.

### 2.5 Preparation of FITC-labeled L100

Fluorescein Isothiocyanate-labeled L100 was prepared using the emulsion extrusion method. In brief, 5 mg of FITC was dissolved in 5 mL of dichloromethane to prepare the oil phase. Eudragit L100-55 was dissolved in NaOH (1 M) to prepare the aqueous phase. Then, adding 0.25 mL of the oil phase to 10 mL of the aqueous phase and subjecting the mixture to an ice bath cooling for 30 s using an ultrasonic disruptor (LAWSON, UP-400, China) to form the O/W emulsion. Under ultrasonic conditions, injecting the O/W emulsion through a needle with an inner diameter of 0.25 mm into the acetic acid (0.05 M) using a syringe pump (Longer, TJ-3A, China) at a flow rate of 2 mL/h, while maintaining the pH < 5 with glacial acetic acid. After forming the FITC-L100 suspension, the organic solvent in the suspension was evaporated using a rotary evaporator. Afterward, the sediment was centrifuged at 8000 rpm for 3 min and washed thrice by distilled water with pH < 5. Finally, freeze-drying the obtained FITC-L100 nanoparticles overnight using a lyophilizer.

### 2.6 Characterization of EcN, EcN@T and EcN@TL

Firstly, the particle size and zeta potential of EcN, EcN@T, and EcN@TL were determined by DLS (Malvern, Zetasizer Pro, England). Then the morphology of bacteria was observed using transmission electron microscopy (TEM) and scanning electron microscopy (SEM). Briefly, after fixing bacteria with 30 % glutaraldehyde solution for 30 s, a drop of bacterial solution was added onto a carbon-coated copper mesh, followed by staining with a 2 % sodium phosphotungstate solution for 30 s and drying, then imaged by TEM (JEM, JEM-F200, Japan). The distribution of elements on the surface of EcN@T was observed using the Energy Dispersive Spectrometer (EDS) module of TEM. For SEM, bacteria were immersed in 5 mM PIPES buffer containing 2 % glutaraldehyde overnight for immobilization. After successive dehydration with different concentrations of ethanol, a drop of bacterial solution was added to coverslips and dried, and imaged by SEM (Thermo, Scientific Verios 5 UC, USA). Subsequently, EcN@TL was characterized by Laser Scanning Confocal Microscopy (LSCM, Zeiss, LSM800, Germany). In short, EcN@T was added to Hoechst 33342 (5 μg/mL)/BSA-Cy5.5 (5 μM) mixture and vibrated for 5 min followed by 15 min of incubation. Finally, the FITC-labeled L100 was prepared and coated onto the EcN@T. The labeled bacteria were washed twice with PBS after each labeling procedure. *Lactobacillus paracasei* were characterized using the same procedures.

### 2.7 Cell growth curves and viability assays

Bare EcN, EcN@T, and EcN@TL (500 μL) were added to LB liquid medium (10 mL) and incubated, and their values of absorbance at 600 nm (OD_600_) were monitored hourly using a UV–visible spectrophotometer (Lengguang Technology, UV7600, China), uncoated EcN was applied as a control. Cell viability was detected by Cell Counting Kit-8 (CCK8, MCE, China), briefly, 20 μL of each group of bacterial fluids, 180 μL of LB liquid medium, and 20 μL of CCK8 solution were inoculated into a 96-well plate, respectively, and incubated in 37 °C, the values of OD_450_ were detected at 1 h and 2 h by Microplate reader (Agilent, Biotek SynergyH1MF, USA).

### 2.8 Auto-detachment of the TA coating

EcN@T was pre-prepared, resuspended in PBS, and incubated at 37 °C. After 3 h, another batch of EcN@T was prepared using the same procedure. Then, Hoechst 33342 and Cy5.5 were simultaneously added to the two bacterial suspensions, followed by 5 min vibration and 15 min of incubation. Finally, after the double rinsing of PBS, the durability of the TA coating was observed using Laser Scanning Confocal Microscopy.

### 2.9 Enzyme activity and antioxidant assays

Catalase activity was assayed by the ammonium molybdate method (Njjcbio, China), SOD activity was assayed by the WST-8 method (Beyotime, China), and the antioxidant properties were demonstrated by the total antioxidant capacity (Beyotime, China) and the survival of probiotics after H_2_O_2_ (15 mM) treatment. For enzyme activity assays, EcN@Te was respectively mixed with H_2_O_2_ or xanthine oxidase/WST-8 solution, and ammonium molybdate was added after incubation, absorbance was detected by UV spectrophotometer. Taking EcN@T as a negative control and enzyme solutions as a positive control to calculate adsorption rates. For antioxidant properties, equal amounts of EcN, EcN@T and EcN@Te precipitates were resuspended in 500 μL of 15 mM H_2_O_2_ and incubated for different periods of time. After the incubation, the solutions were centrifuged and resuspended in 500 μL of live and dead cell fluorescent probes or PBS. Finally, the antioxidant properties of EcN in different groups were observed using LSCM or colony-forming unit (CFU) counting.

### 2.10 Harsh environment resistance assays

Equal amounts of EcN, EcN@T, and/or EcN@Te and/or EcN@TL were separately added to simulated gastric fluid (SGF, pH 2.0, consisting of 0.85 % NaCl and 1 % pepsin), simulated intestinal fluid (SIF, pH 6.8, consisting of 0.68 % KH_2_PO_4_ and 1 % trypsin), 0.1 mg/mL of antibiotics (ampicillin, gentamicin, ciprofloxacin, tobramycin, sulfate neomycin, norfloxacin, levofloxacin), and incubated at 37 °C at 150 rpm. Each group of the bacterial solution was removed and washed with ddH_2_O at specific time points, and 100 μL of liquid was aspirated on LB agar plates after serial 10-fold dilutions of specific multiplicity, colonies on the plates were recorded after 24 h of proliferation in the incubator. The survival of LP was investigated by the same procedures.

### 2.11 Animals

The 8-week-old male Balb/c mice used in this study were purchased from Beijing Vital River Laboratory Animal Technology Co., Ltd. and Guangzhou Chashi Ruihua Biotechnology Co., Ltd.

### 2.12 In vivo biosafety assessment

Mice were administrated with 5 × 10^7^ CFUs of EcN@TeL or saline for ten days and their body weight was recorded daily. Their blood and major organs were collected, after measurement for weight and length, organs were soaked into 4 % paraformaldehyde for fixation, then hematoxylin-eosin (H&E) staining and imaging were conducted after dehydration, paraffin embedding, and sectioning. Changes in various cytokines in serum and blood were detected by an automatic biochemical analyzer and an automatic hematology analyzer.

### 2.13 In vitro mucosal adhesion evaluation of EcN@T

The cell membrane fluorescent probe DIR (5 μM) was incubated with 500 μL of EcN (5 × 10^7^ CFUs) for 30 min. The freshly colonic tissues were divided into about 1.5-cm segments and longitudinally slit to expose the internal mucosa and co-incubated with equal amounts of EcN or EcN@T (labeled with DIR) in PBS for 1 h at a slightly up-down shaking (30 rpm). Subsequently, colonic segments were soaked in 50 mL of deionized (DI) water twice with allegretto shaking (80 rpm) to remove the unadhered bacteria. Finally, the fluorescence intensity of the colons was observed and quantified using an *in vivo* imaging system (IVIS, Caliper, IVIS Kinetic, USA), and the amount of attached EcN was determined by CFU counting.

### 2.14 In vivo colonization and bioavailability evaluation

After the gavage of 5 × 10^7^ CFUs of EcN, EcN@L, EcN@T, and EcN@TL (labeled with DiR, expressing resistance to ampicillin), probiotics in the GI tract as well as in isolated intestines were observed and quantified using IVIS at indicated time points. To investigate whether the TA-L100 coatings could improve the colonization capacity of EcN, we collected fecal samples and intestinal tissues from different regions at specific time points after gavage, which were added with 10-fold weight of PBS and homogenized, and 100 μL of suspension was then taken and spread on selective LB agar plates after serial 10-fold dilutions, and the colonies were counted after overnight incubation.

### 2.15 The therapeutic effect of probiotics on DSS-induced colitis

To evaluate the *in vivo* synergistic therapeutic effect of EcN@TeL, DSS-induced mouse colitis models were established. In brief, Balb/c mice were given DI water containing 3 % DSS for 7 days to induce acute colitis. Afterward, the mice were given 5-ASA (200 mg/kg), bacterial suspension (EcN, EcN@L, EcN@TL, EcN@TeL, 5 × 10^7^ CFUs), and the same volume of saline *via* gavage daily until the 11th day. Body weight changes, fecal traits, and fecal occult blood test of mice were recorded daily, DAI is the summation of scores of each part (Table S1). The mice were euthanized on day 12, and their colons, blood, spleens, and intestinal feces was collected. After measuring the colonic length, 0.5–1 cm of the distal colon segments were excised and dipped in 4 % paraformaldehyde solution for fixation and sent for histopathology assessment. The serum was isolated by centrifugation at 3000 rpm for 20 min and the internal cytokines associated with inflammation were detected using ELISA. The colonic excrement was immersed in liquid nitrogen immediately and sent for sequencing analysis.

### 2.16 Prophylactic effect of probiotics against DSS-induced colitis

Balb/c mice were given DI water containing 3 % DSS for 7 days. Saline, 5-ASA (200 mg/kg), and bacteria suspension (EcN, EcN@L, EcN@TL, EcN@TeL, 5 × 10^7^ CFUs) were administrated for 10 days, with the saline group serving as a blank control. Body weight changes, fecal traits, and fecal occult blood test of mice were recorded daily, DAI is the summation of scores of each part (Table S1). The mice were euthanized on day 11, and the isolated serum and colonic excrement were steeped in liquid nitrogen for backup. The colonic length was measured, and the distal colons were sampled and dipped in 4 % paraformaldehyde solution for fixation and histological analysis.

### 2.17 Histopathology assessment

The damage extent of fixed tissues was appraised by H&E, immunohistochemistry (IHC), and immunofluorescence (IF) staining. Briefly, isolated tissues were fixed in 4 % paraformaldehyde, embedded in paraffin, sliced, and stained with H&E. The slides were scanned for cellular structures using a microscope (Leica, DM2000 LED, Germany). Each colonic section was blindly scored by trained pathologists for the degree of tissue damage according to Table S2 (including ulceration, epithelial cell changes, and degree of inflammatory infiltration). For IHC, tissular antigens were repaired by hyperthermia and then respectively incubated with serum, and primary and secondary antibodies (Servicebio, China). Brownish-yellow positive cells were observed because of the reaction between peroxidase and 3,3′-diaminobenzidine (DAB), and the nuclei were redyed to blue by hematoxylin. For IF, sections were subjected to antigen repair, serum closure, primary and secondary antibody incubation, and tyramide signal amplification (TSA), respectively, then viewed and imaged by ortho-fluorescence microscopy (Nikon Eclipse C1, Japan). The positive areas and their fluorescence intensity were calculated and analyzed using image J and corrected by areas. Briefly, the positive areas in IHC staining sections were computed using the IHC Tool Box, and the control group was taken as control to analyze the relative positive areas with a threshold in the range of 130–180. The color channels of IF staining images were split and the fluorescence intensity in the channel of interest was analyzed with a threshold within the scope of 50–75.

### 2.18 Enzyme-linked immunosorbent assays

Enzyme-linked immunosorbent assays (ELISA, X-Y Biotechnology and Jiangsu Meimian Industrial Co., Ltd) were performed to quantify the concentration of ROS and the secretion of UC-related cytokines, and all procedures involved were completed in a low-temperature environment. For blood samples, the separated blood was rested for 30 min and then centrifuged at 3000 rpm for 20 min, and the supernatant serum was aspirated for backup. For tissue samples, tissues were added with a 5-fold weight of ice PBS and oscillated by a tissue grinder (SCIENTZ-24, China) at 60 Hz for 1 min. After the centrifugation at 12000 rpm for 10 min, the supernatant was aspirated for backup. According to the ELISA kit instructions, standards and samples were inoculated in a microtiter plate to bind specifically to the antigen, unbound factors were washed away, after the addition of detection antibody, horseradish peroxidase, and enzyme substrate, the concentration of target proteins was indirectly detected by microtiter plate detector.

### 2.19 Real-time fluorescence quantitative PCR (qPCR)

The mRNA expression levels of tumor necrosis factor alpha (*Tnf-α*), transforming growth factor beta (*Tgf-β*), interleukin 1 beta (*Il-1β*), interleukin 6 (*Il-6*), interleukin 10 (*Il-10*), and mucin 3 (*Muc3*) was detected by qPCR. The housekeeping gene, beta-actin (*Actb*), was used as a control, and the target genetic fluorescence values were analyzed using the 2^−ΔΔCT^ method and indicated by relative expression in comparison to the control gene. According to the kit instructions, colon tissues were pulverized using a tissue grinder, the total RNA was precipitated by isopropanol, and its integrity was detected using agarose gel electrophoresis. Then, reverse transcription to cDNA was performed using the following procedure: 42 °C for 2 min, followed by 50 °C for 15 min and 85 °C for 5 s. The qPCR system consisted of enzyme-free water, primers (Sangon Biotech, China, Table S3, 0.3 μM), cDNA (≤200 ng), and fluorochrome, was amplified by the following procedure: 95 °C for 30 s followed by 40 cycles of 95 °C for 5 s and 60 °C for 45 s.

### 2.20 16S rRNA sequencing analysis

Colonic feces of mice were collected on day 11 or 12 and immersed in liquid nitrogen immediately, subsequently sent to Shanghai Majorbio Bio-Pharm Technology Co., Ltd. for sequencing analysis. Genetic amplification was performed using universal primers 338F (ACTCCTACGGGGAGGCAGCAG) and 806R (GGACTACHVGGGTWTCTAAT) designed to target the V3-V4 region of the bacterial 16S rRNA gene by ABI GeneAmp®9700 PCR thermal cycler (ABI, CA, USA). Sequencing of amplicons was performed using the Illumina MiSeq PE300 platform. Sequences containing ambiguous bases or more than 200 bp in length or with an average quality score of less than 20 were removed at the end of sequencing. The sequences were clustered into operational taxonomic units (OTUs) with 97 % sequence homology using VSEARCH (1.9.6). After categorizing all OTUs, species richness and evenness within the samples were obtained by Alpha diversity calculation and Venn diagram. In addition, multiple sequence comparisons of OTUs were performed, and high-dimensional class comparisons were performed *via* Principal Component Analysis (PCA) and Linear discriminant analysis Effect Size (LefSe), to explore the differences in community structure among different samples or groups. All data obtained were analyzed on the Majorbio Cloud platform (https://cloud.majorbio.com/).

### 2.21 Extraction and quantification of SCFAs

To further evaluate EcN@TeL's beneficial role in the intestine, we extracted and quantified the SCFAs in mouse feces. Briefly, standard stock solutions of SCFAs (400 mg/mL) and internal standard solution (2-ethyl-butyric acid, 10 mg/mL) were prepared using methanol as solvent. The standard stock solutions were diluted into working solutions of 1, 5, 20, 100, 200, 300, 500, and 800 μg/mL using methanol and intrinsic standard solution, respectively.

The fecal samples (1 mg/mL, containing 60 mM HCl) were crushed using a tissue grinder, sonicated for 30 min in an ice bath, let stand for 30 min, and then centrifuged at 13000 g for 10 min. Methanol (2 ml) was added to the separated supernatants, mixed well and then centrifuged at 13000 g for 10 min. Anhydrous sodium sulfate (0.5 g) was added to the separated supernatants, mixed well and then centrifuged at 13000 g for 10 min. The supernatants were filtered through a 0.22 μm organic-phase microporous membrane, and then the intrinsic standard application solution (100 μg/mL) was added.

Short-chain fatty acids in colon feces were detected using gas-phase chromatography (Agilent 6890N, USA). The concentration of the standard solution was used as the horizontal coordinate, and the ratio of the peak area of standard to the peak area of internal standard was used as the vertical coordinate to make standard curve, and the SCFA concentration was calculated from the corresponding standard curve.

### 2.22 Statistical analysis

Data differences were analyzed using GraphPad Prism 9.0.0 with one-way and two-way analysis of variance (ANOVA), followed by Tukey's Multiple Comparison Test for between-group means. All data were presented as mean ± standard error of mean (SEM). Statistical significance is shown below: ∗*P* < 0.05, ∗∗*P* < 0.01, ∗∗∗*P* < 0.001, ∗∗∗∗*P* < 0.0001, ns, not significant.

## 3 Results

### 3.1 Preparation and characterization of coated probiotics

**Fig. 1:**
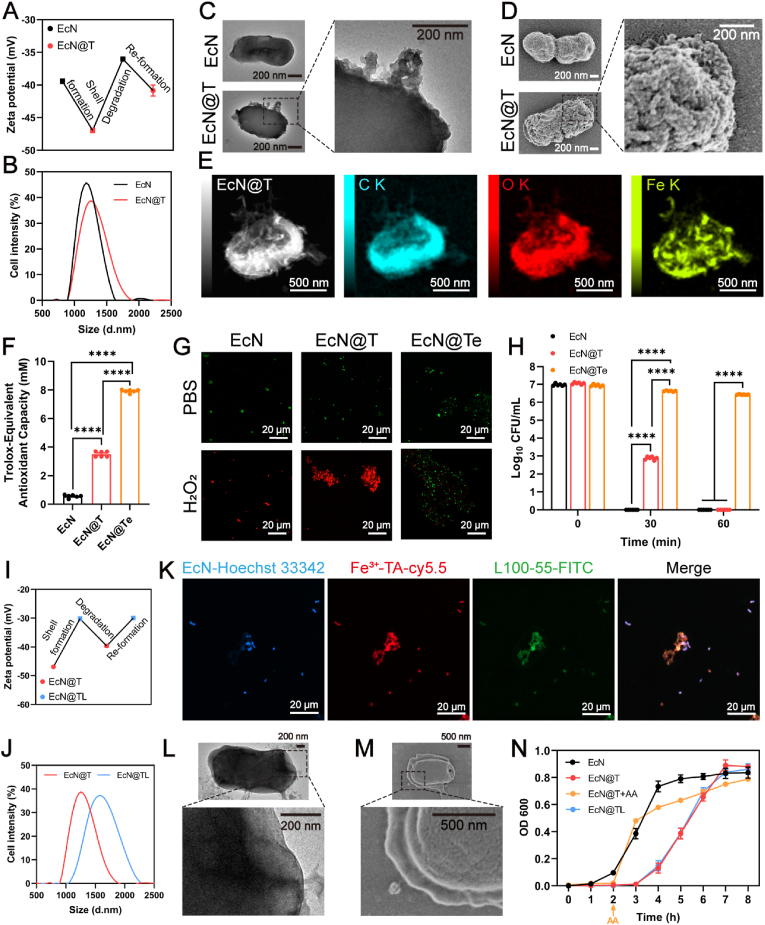
Characterization and functional verification of enveloped EcN. (A) Changes in zeta potential during the encapsulation and decapsulation of TA (n = 6). (B) Cell size of EcN and EcN@T. (C–D) Classic TEM and SEM images of EcN and EcN@T, as well as the local feature images of EcN@T. Scale bars, 200 nm. (E) Typical EDS images of EcN@T. Scale bars, 500 nm. (F) Total antioxidant capacity assays of EcN, EcN@T, and EcN@Te. (G) Typical LSCM images of EcN, EcN@T, and EcN@Te labeled with live/dead probes with or without 15 mM H_2_O_2_ treatment for 30 min. Scale bars, 20 μm. (H) Bacterial counts after treatment with 15 mM H_2_O_2_ for 0–60 min (n = 6). (I) Variation of zeta potential during the encapsulation and decapsulation of L100 (n = 6). (J) Cell size of EcN@T and EcN@TL. (K) Typical LSCM images of EcN@TL. Scale bars, 20 μm. (L–M) Representative TEM and SEM images of EcN@TL, as well as their local magnification. Scale bars, 200 nm for TEM and 500 nm for SEM. (N) Growth curves of EcN, EcN@T, EcN@T + AA, and EcN@TL (n = 3). Data were presented as mean ± SEM, statistical analysis was performed using one-way ANOVA for (F) and two-way ANOVA for (H), ∗∗∗∗*P* < 0.0001.

To demonstrate the proof of principle, EcN, a well-known probiotic recognized for its capability to modulate host-microbiota homeostasis and collaborate with other probiotics in enzyme production, local immunity, and digestion [40], was chosen as the model bacterial strain. Food and Drug Administration (FDA)-approved safe materials, TA and Fe^3+^, were chosen as coating materials on account of their extraordinary chelation capability in wrapping bacterial cells, together with the outstanding adhesiveness of TA provided by its pyrogallol and catechol groups (Fig. S1A) [41]. FeCl_3_ and TA were sequentially mixed with EcN *via* gentle shaking and the entire coating process could be finished within 1 min. The coated EcN (EcN@T) turned black, while the uncoated EcN remained pale (Fig. S2A). What's more, the coating could be degraded in response to ascorbic acid (AA), enabling its arbitrary disintegration as requested (Fig. S2B–C). The zeta potential changed from −39.4 mV to −47.0 mV attested the successful envelopment of Fe^3+^-TA shells, and the surface charge of EcN showed a sawtooth-like change during the repeated artificial encapsulation and dissociation of Fe^3+^-TA shells with the additional introduction of Fe^3+^, TA, and AA solutions, separately, further clearly demonstrating the shell's formation and degradation process (Fig. 1A). In addition, the TA coating increased the EcN size by 80 nm (Fig. 1B), and the jagged-like shell around the surface of EcN could be observed in TEM and SEM images (Fig. 1C–D), which were parallel to the particle size results, further confirming that individual cells, not cell aggregates, were encapsulated within TA shells. To further validate the successful encapsulation, we analyzed the elemental composition of the EcN@T surface using EDS. The coating network-related distribution of iron was observed (Fig. 1E), further denoting the existence of Fe^3+^-TA film on EcN. In addition, TA has been certified to have strong absorption in the ultraviolet (UV) wavelength range [32], therefore, we further confirmed its encapsulation using UV resistance experiment. As shown in Fig. S3, all naked EcN died after 30 min of UV irradiation, whereas EcN@T still maintained high physiological activity, proving the successful encapsulation and the UV-protective property of TA nanocoating.

Metal–phenolic networks (MPNs) exhibit strong interactions with proteins [42]. Phenolic groups are known as excellent hydrogen donors, capable of forming hydrogen bonds with the carbonyl and amino groups of proteins [43,44]. We hypothesized that antioxidant enzymes could be embedded within the latticed shell, effectively scavenging the abnormally proliferated ROS, and alleviating the symptoms of UC (Fig. S1B). To verify our conjecture, we incubated EcN@T with CAT and SOD for 60 min, the enzymes could promote the proliferation of EcN in the absence of H_2_O_2_ (Fig. S4), while the enzyme activities simultaneously decreased as time passed (Fig. S5). Subsequently, 15 mM H_2_O_2_ was added to the system to explore the optimal unit of enzymes (Fig. S6A). As shown in Fig. S6B–C, cells in the EcN@T group were all killed after exposure to H_2_O_2_, and the decreased enzyme activities resulted in a reduced protective effect of EcN@T against H_2_O_2_ as embedding time increased, whereas the 10-min adsorption of antioxidant enzymes could greatly improve the viable probiotics up to 10^6^ CFU/mL. Notably, embedding CAT and SOD simultaneously within TA coatings exhibited the best-protecting effects on cells (Fig. S6D), which attested to our hypothesis. Moreover, EcN@Te exhibited remarkable resistance to H_2_O_2_ after PBS washing and 1–4 h of storage in PBS (Fig. S7), demonstrating that the antioxidant enzymes with optimal concentrations (10-min absorption, CAT: 210 U, SOD: 200 U) could be adsorbed by MPNs and remain stable. The average absorption rate of CAT and SOD could reach 36.88 % and 76.11 % within 10 min, respectively (Fig. S8), and the total antioxidant capacity of EcN@Te after enzyme loading was increased by 126.5 % compared to EcN@T (Fig. 1F). To further confirm the formation of the catalytic layer, we used live/dead fluorogenic indicators to label EcN in different groups after H_2_O_2_ treatment, and the results were shown in Fig. 1G, based on typical LSCM images, massive mortality was observed in the EcN and EcN@T groups, while only a few cells died in the EcN@Te group. To further validate the above results, we counted the survival of EcN in different groups (Fig. 1H). All EcN and EcN@T cells died after 60 min of H_2_O_2_ treatment, while EcN@Te maintained a level of 10^6^ CFU/mL throughout. These results not only demonstrated the successful construction of the TA-enzyme loading platform but also revealed its powerful antioxidant capacity.

Finally, we further coated EcN@T with L100 to overcome the first threshold (aggressive acidic and digestive gastric fluid) faced by probiotics during oral delivery, and the characterization experiments were performed (Fig. S1C). As shown in Fig. 1I–J, a sawtooth-like polyline appeared during the encapsulation and degradation of L100, which increased the size of EcN@T by about 300 nm, indicating the successful formation of L100 shell on the surface of EcN@T. To visualize the formation of the TA and L100 shells, EcN was labeled with Hoechst 33342 (blue), Fe^3+^-TA coating was labeled with Cy5.5 (red), and L100 shell was labeled with FITC (green), the signals of each fluorescence pathway were observed by LSCM. As shown in Fig. 1K, the green bacteria, red Fe^3+^-TA coatings, and green L100 shells were co-localized with high efficiency, indicating that cells were individually coated with TA-L100 shells. In addition, the L100 shell brought a smooth outward shell different from that of TA (Fig. 1L–M), which further confirmed the existence of L100 on the surface of EcN@T. To examine whether the coating process could affect the viability of cells, we further evaluated the growth curves before and after coating. As shown in Fig. 1N, after the formation of TA, the growth curves of EcN@T displayed a growth delay of about 3 h. However, upon the addition of AA, the encased cells immediately regained growth activity and switched to a dividing mode. Combining the live/dead fluorescence plots of EcN@T, the coating process did not affect cell viability, and the cells might enter a temporary ‘dormant’ state while being encapsulated. Delightfully, after the incubation of EcN@T in PBS for 3 h, the fluorescence of Fe^3+^-TA was diffuse and did not localize to EcN (Fig. S9), indicating that TA shell could automatically detach after 3 h and have enough time to promote the attachment of probiotics to the gut, and then auto-degrade in the intestine without extra use of AA. In addition, the cell counting kit assay also confirmed that the coating processes made a negligible impact on cellular viability (Fig. S10). In addition, the characterization of LP exhibited parallel results, further demonstrating the successful modularity of the coating strategy (Fig. S11).

### 3.2 Protection effect of shells against GI tract stress

**Fig. 2:**
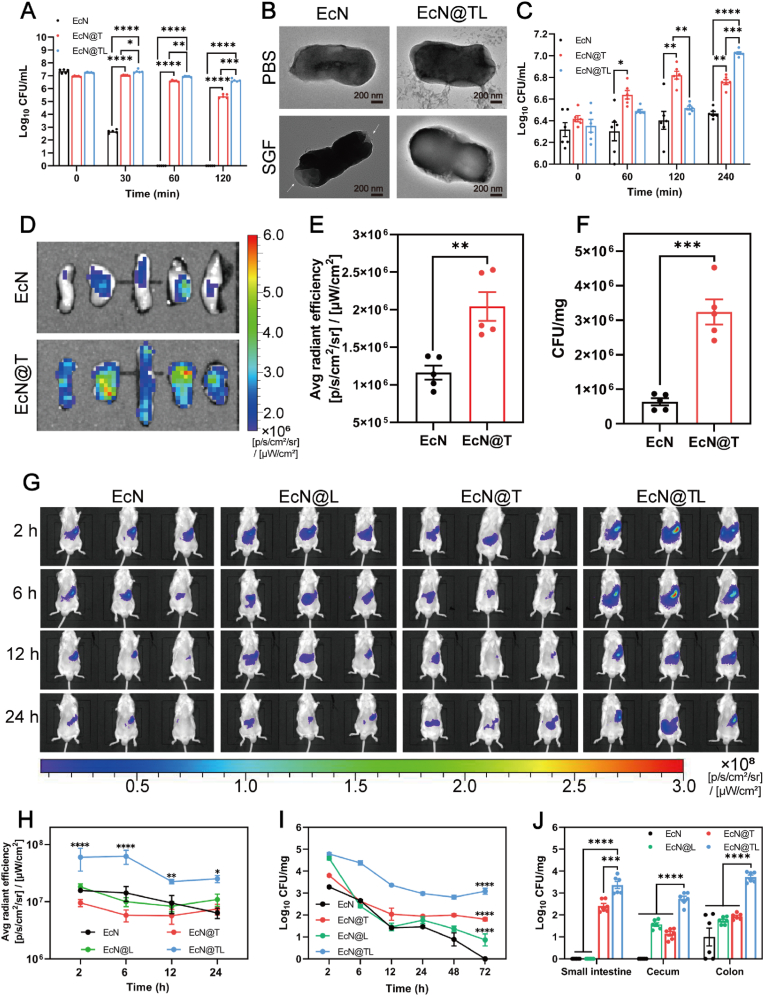
Protection effect of shells against GI tract stress *in vitro* and *in vivo*. (A) Survival of EcN, EcN@T, and EcN@TL after digestion by SGF for 0–120 min (n = 6). (B) Classic TEM images of EcN and EcN@TL after treatment with PBS or SGF for 120 min. Scale bars, 200 nm. (C) Bacterial counts of EcN, EcN@T, and EcN@TL after treatment with SIF for 0–240 min (n = 6). (D) The isolated colon segments imaged by IVIS after co-incubation with EcN and EcN@T labeled by DIR with fluorescence excitation (n = 5). (E) Quantitative analysis of fluorescent signals based on (d) (n = 5). (F) Bacterial counts of EcN and EcN@T attached to the colons. (G) Classic IVIS images of mice at different time points after oral gavage of EcN, EcN@L, EcN@T, and EcN@TL. (H) Quantitative evaluation of fluorescent signals of EcN, EcN@L, EcN@T, and EcN@TL at different time points (n = 3). (I) Quantitative analysis of living EcN in feces of mice at different time points (n = 6). (J) The viable bacterial counts in the small intestine, cecum, and colon sampled at 24 h after gavage of EcN, EcN@T, EcN@L, and EcN@TL. (n = 6). Data were presented as mean ± SEM, statistical analysis was performed using one-way ANOVA for (E–F) and two-way ANOVA for (A), (C), (H–J), ∗*P* < 0.05, ∗∗*P* < 0.01, ∗∗∗*P* < 0.001, ∗∗∗∗*P* < 0.0001, ns, not significant.

Physiological activity and on-demand release of EcN in the intestine are crucial for its therapeutic efficacy on colitis. Therefore, simulated digestion tests of coated probiotics were performed *via* incubating native and coated probiotics with SGF and SIF to investigate whether the shells could improve the *in vitro* bioavailability of probiotics (Fig. S12). As shown in Fig. 2A–B, native EcN died apace within 60 min upon exposure to SGF, which were subjected to morphological changes, cell rupture, and exudation of cell contents (white arrows), while EcN@T maintained a high survival rate, indicating that TA shell could withstand the degradation of SGF to some extent. It's noteworthy that the cell viability of EcN@TL was patently higher than that of EcN@T due to the resistance of L100 to strong acid and deformation. Next, we evaluated the accurate release properties of coated probiotics. As shown in Fig. 2C, after the depolymerization of L100 triggered by neutral pH and the exfoliation of TA, the CFUs of EcN in the EcN@T and EcN@TL groups were significantly higher than that of unencapsulated EcN, suggesting that EcN@TL could be on-demand reactivated and respond to the surroundings more quickly. Then we sequentially utilized the above digestive fluids and H_2_O_2_ to simulate an inflamed GI tract. To resist H_2_O_2_, we loaded antioxidant enzymes within the shells. As shown in Fig. S13, the viability of EcN within the EcN@TeL group was 14.75 times higher than that of the EcN@TL group after sequentially treating with SGF and SIF containing H_2_O_2_, indicating that the enzymes indeed could protect cells from severe oxidative stress. In addition, considering antibiotics are usually administrated against pathogens during colitis treatment, which will cause a mass die-off of probiotics. Therefore, we further evaluated whether EcN@TL could resist the damage of six broad-spectrum antibiotics. As expected, antibiotics killed more than 99 % of native EcN, but EcN@TL was able to maintain a high survival rate (Fig. S14), opening up the possibility of combining coated probiotics with antibiotics to attain better therapeutic results. In addition, the *in vitro* resistance of LP to harsh environments showed similar trends as EcN (Fig. S15), supporting the broad applicability of the TA-L100 coating strategy for protecting various probiotics in the GI tract.

To investigate whether the outstanding protective effect of TA-L100 shells could improve the bioavailability of probiotics, we further evaluated its bioavailability *ex vivo* and *in vivo* (Fig. S12). Polyphenol-metal ion complexes can greatly enhance the *in vivo* bioavailability of probiotics due to their strong adhesion properties, which arise from the formation of hydrogen bonds, covalent bonds, and/or π-π stacking interactions between pyrogallol/catechol and substrates [[45], [46], [47]]. To verify the adhesion of the TA shell, we co-cultured freshly isolated colonic segments with EcN or EcN@T, where EcN was labeled with a DIR fluorescent probe, and the fluorescence intensity of tissues was observed using IVIS. As shown in Fig. 2D, there was a noticeable distribution of EcN@T in the colons, while the distribution of EcN was unobvious, and the fluorescence intensity of the EcN@T-attached colons were 1.75 times higher than that of the EcN-attached colons (Fig. 2E). Then the number of attached EcN was calculated using LB ager plates with ampicillin, as shown in Fig. 2F, the number of EcN adsorbed on colons in the EcN@T group is 6.03 times that of the EcN group, proving that the TA shell could help EcN to cling and significantly augment its adhesive effect in the colon. Next, the *in vivo* bioavailability of EcN in diverse groups after gavage was observed using IVIS. As shown in Fig. 2G–H, the fluorescence intensity of mice in EcN@T and EcN@L groups was similar to that of native EcN group, while the highest fluorescence intensity was exhibited in EcN@TL group, indicating that merely TA or L100 offered limited reinforcement to EcN, the strong adhesion of TA and the protective property of L100 were indispensable for increasing the adhesion of EcN in intestines. What's more, similar results appeared in typical intestinal fluorescence images (Fig. S16). To further quantify the survival of EcN in the GI tract, LB selective plates were utilized to detect the amount of living EcN from different groups in the intestinum tenue, cecum, colon, and excreta. Mouse feces were collected at 2, 6, 12, 24, 48, and 72 h after a single gavage, as shown in Fig. 2I, the colony units of EcN@L and EcN@TL groups were similar at 2 h, demonstrating that the L100 coating effectively limited the loss of coated EcN through gastric digestion and increased its brief exposure in the intestine. However, EcN@L gradually lost due to the absence of enhanced adhesion. Moreover, EcN@T and EcN groups without L100 coatings showed poor survival. Only the EcN@TL group maintained the highest colony count and persisted for more than 72 h, the colonization of the EcN@TeL group was 25.2 times and 256.2 times higher than that of the EcN@T and EcN@L groups, respectively. Indicating that the TA-L100 coatings could effectively prolong the transit time of EcN in intestines and increase its colonization. In addition, after 24 h of administration, EcN in the EcN@T and EcN@TL groups were widely distributed throughout the entire intestines, while EcN and EcN@L only emerged in the distal intestines, further confirming the adhesive ability of TA coating. Moreover, the number of alive EcN in the EcN@TL group in all intestines was significantly surpassed that in the native EcN, EcN@T, and EcN@L groups (Fig. 2J), which certified that TA and L100 could synergistically increase the colonization ability of EcN, leading to a decrease in its early loss through faces and an increase in the total exposure of the maximum concentration, without hindering the subsequent growth of probiotics or their interaction within GI tract. In summary, TA-L100 layers could significantly enhance the *in vivo* bioavailability of EcN.

### 3.3 *In vivo* biosafety assessment of EcN@TeL

**Fig. 3:**
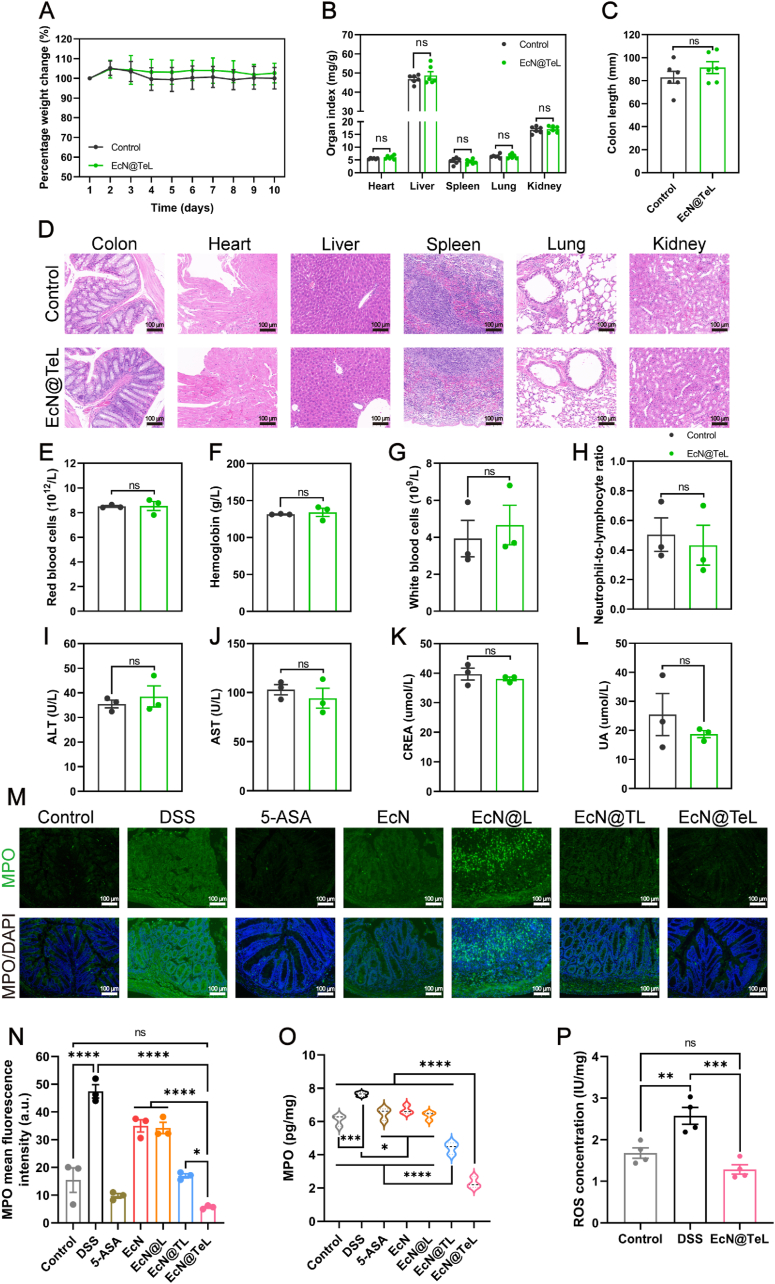
*In vivo* biosafety assessment of EcN@TeL and its palliative effect on colonic ROS. (A) Percentage changes in body weight of mice after gavage with saline or EcN@TeL for 10 days (n = 6). (B) Major organ indices of mice treated with saline or EcN@TeL (n = 6). (C) Quantitative analysis of colon lengths in different treatment groups (n = 6). (D) Classical histological section images of major organs stained with H&E. Scale bars, 100 μm. (E–H) Blood biochemistry examination including red and white blood cell counts, hemoglobin concentration, and neutrophil-to-lymphocyte ratio (n = 3). (I–J) Liver function tests include the amount of ALT and AST (n = 3). (K–L) Kidney function test including the concentration of creatinine (CREA) and uric acid (UA) (n = 3). (M–N) Classic IF images of colon sections based on MPO and their quantitative analysis of mean fluorescence intensity (n = 3). Scale bars, 100 μm. (O) MPO concentration in colons (n = 3). (P) ROS activity in colons (n = 4). Data were presented as mean ± SEM, statistical analysis was performed using two-way ANOVA for (A–B), and one-way ANOVA for (C), (E–L), (N–P), ∗*P* < 0.05, ∗∗*P* < 0.01, ∗∗∗*P* < 0.001, ∗∗∗∗*P* < 0.0001, ns, not significant. (For interpretation of the references to color in this figure legend, the reader is referred to the Web version of this article.)

Before conducting *in vivo* experiments, we first systematically evaluated the safety of oral administration of EcN@TeL for 10 days. As expected, the mice did not show abnormal signs after gavage of EcN@TeL, and their body weights, various organ indices, and colon lengths were almost the same as those of the control group (Fig. 3A–C). There is no abnormal inflammatory infiltration or pathological alterations showed in pathological sections of the major organs (Fig. 3D), demonstrating the cellular safety of EcN@TeL. Furthermore, the mice did not have any significant changes in their blood biochemistry parameters including erythrocytes, hemoglobin, leukocytes, and neutrophil-to-lymphocyte ratios (Fig. 3E–H). The liver function parameters including alanine aminotransferase (ALT) and aspartate aminotransferase (AST), and the renal function parameters including creatinine (CR) and uric acid (UA) were within the normal ranges (Fig. 3I–L), indicating that EcN@TeL had no organ toxicity and did not cause any adverse reactions. Taken together, EcN@TeL exhibited benign biosafety *in vivo*.

### 3.4 Palliative effect of EcN@TeL on colonic ROS

Encouraged by the excellent biosafety performance of EcN@TeL, we further evaluated its therapeutic effect on colitis. There is clear evidence that ROS accumulation may indicate or lead to irreversible oxidative events that cause permanent damage to biomolecules such as DNA, lipids, and proteins, ultimately leading to cellular death and/or the potential development of disease [48,49]. ROS accumulation not only damages intestinal epithelial cells, but also inhibits the colonization of beneficial bacteria in inflammatory areas [50,51]. Considering the catalytic property of EcN@TeL, we first confirmed its ROS scavenging function *in vivo*. We utilized 3 % dextran sulfate sodium salt (DSS) to continuously feed mice for 7 days to build colitis models, the control group was given the same volume of distilled water. The same volume of saline, different treatments of EcN and 5-aminosalicylic acid (5-ASA) were given to mice *via* gavage for 5 days after the successful modeling (EcN, 5 × 10^7^ CFU/d, 5-ASA, 250 mg/kg/d) (Fig. S17A). As shown in Fig. S17B–C, the colon lengths of mice in the DSS-treated group were significantly shorter than that of control and EcN@TeL groups, which reflected the therapeutic potential of EcN@TeL. As shown in representative IF-stained sections, myeloperoxidase (MPO) was overproduced in the colons of colitic mice (Fig. 3M), which may incur adverse effects during inflammation by indirectly increasing reactive oxygen/nitrogen species (RONS) formation [52]. Encouragingly, all treatment groups could significantly alleviate the above abnormalities, and the MPO levels were lowest in the EcN@TeL group and markedly lower than in the EcN@TL group, suggesting that CAT and SOD might exert an outstanding effect on RONS scavenging. (Fig. 3N). ELISA and IHC staining of colonic tissues further verified the above results (Fig. 3O and Fig. S17D–E). What's more, EcN@TeL significantly lightened the ROS accumulation in inflamed colons, with a slightly lower ROS concentration compared to the control group (Fig. 3P). These outcomes demonstrated that EcN@TeL could effectively restrain the level of MPO, block the production of RONS, and eliminate the excessive ROS within colons with high efficacy.

### 3.5 The therapeutic effect of encapsulated probiotics on UC

**Fig. 4:**
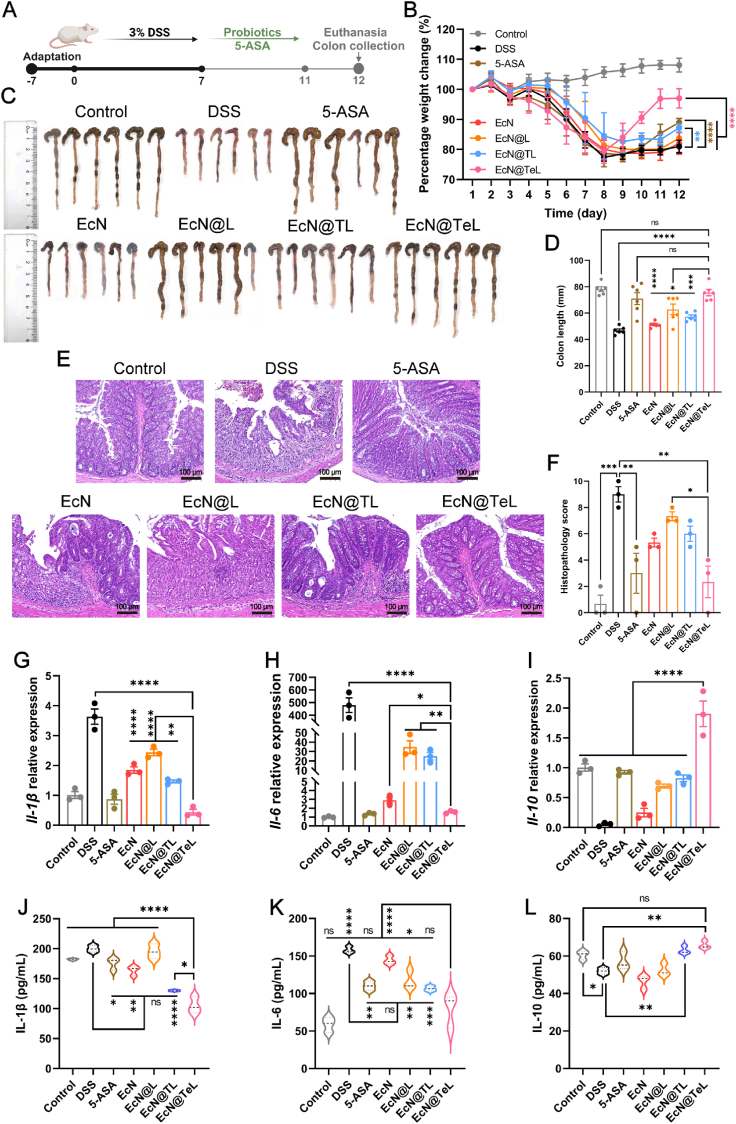
The therapeutic effect of EcN@TeL on ulcerative colitis. (A) Schematic diagram of the experimental procedure. (B) Percentage changes in body weight of DSS-endured mice with different treatments (n = 6). (C) Colon images of mice. (D) Quantitative analysis of colonic length (n = 6). (E) Representative colonic histological section images of mice with different treatments stained with H&E. Scale bars, 100 μm. (F) Quantitative analysis of colonic pathology scores according to Table S2 (n = 3). (G–I) Relative mRNA expression of inflammatory factors including *Il-1β*, *Il-6*, and *Il-10* in colon tissues of mice in different treatment groups (n = 3). (J–L) Concentration of inflammatory factors including IL-1, IL-6, IL-10 in serum of DSS-beared mice in different treatment groups (n = 3). Data were presented as mean ± SEM, statistical analysis was performed using one-way ANOVA for (D), (F–L), and two-way ANOVA for (B), ∗*P* < 0.05, ∗∗*P* < 0.01, ∗∗∗*P* < 0.001, ∗∗∗∗*P* < 0.0001, ns, not significant.

Encouraged by the splendid antioxidant ability of EcN@TeL *in vivo*, we further investigated its therapeutic effects on other aspects of colitis and the experiment procedure was as that of the ROS evaluation (Fig. 4A). As shown in Fig. 4B and Fig. S18, DSS induced weight loss and DAI elevating, which were significantly alleviated after treatment with 5-ASA, EcN@TL, and EcN@TeL, with EcN@TeL demonstrating the best remedial outcome. In addition, the shortened colons of mice were restored to healthy levels by EcN@TeL, and the colon length of the EcN@TeL group was considerably longer than EcN@TL group (Fig. 4C–D), emphasizing that the antioxidant enzymes within EcN@TeL exert a remarkable effect on protecting colons from severe oxidative damage and facilitating colon recovery. Then changes in colons at the microscopic level were observed using H&E staining to appraise the role of coated probiotics in restoring inflamed colonic structure. As shown in representative tissular sections, the colons in the DSS group exhibited pathological changes such as deformation or disappearance of crypts, absence of goblet cells, ulcer production, and infiltration of inflammatory cells, whereas EcN@TeL could significantly reverse the above symptoms and reduce the pathological scores, with no apparent difference from healthy and 5-ASA groups (Fig. 4E–F).

Moreover, pro-inflammatory cytokines are involved in the early response and amplification of the inflammatory immunity, while anti-inflammatory factors limit the inflammatory response. The balance between them determines the trend of inflammatory diseases [53,54]. Therefore, transcriptional and translational assays on colon tissues and serum were performed to explore the role of probiotics in regulating inflammatory cytokines. As expected, compared with the control group, the overproduction of pro-inflammatory factors was observed in the DSS group, with a 3.6-fold, 479-fold, and 1.6-fold increase in *Il-1β*, *Il-6*, and *Tnf-α* mRNA expression, respectively (Fig. 4G–H and Fig. S19A), as well as the downregulation of anti-inflammatory factors, with a 20-fold, 4.4-fold decrease in *Il-10* and *Tgf-β* mRNA expression, severally (Fig. 4I and Fig. S19B). Whereas EcN@TeL could significantly restore the transcriptional level of pro-inflammatory factors and accelerate the expression of anti-inflammatory cytokines, and the subsequent ELISA results further verified the qPCR results (Fig. 4J–L). Noticeably, the effect of EcN@TeL on cytokine modulation had a significant advantage over EcN@TL, further validating the immense potential of enzyme embedding in coordinating cytokines and inactivating inflammation. In a word, EcN@TeL might exert a remedial effect on UC by normalizing immunologic balance and facilitating the restoration of colonic structure.

### 3.6 Repair effect of coated probiotics on UC-induced intestinal barrier dysfunction

**Fig. 5:**
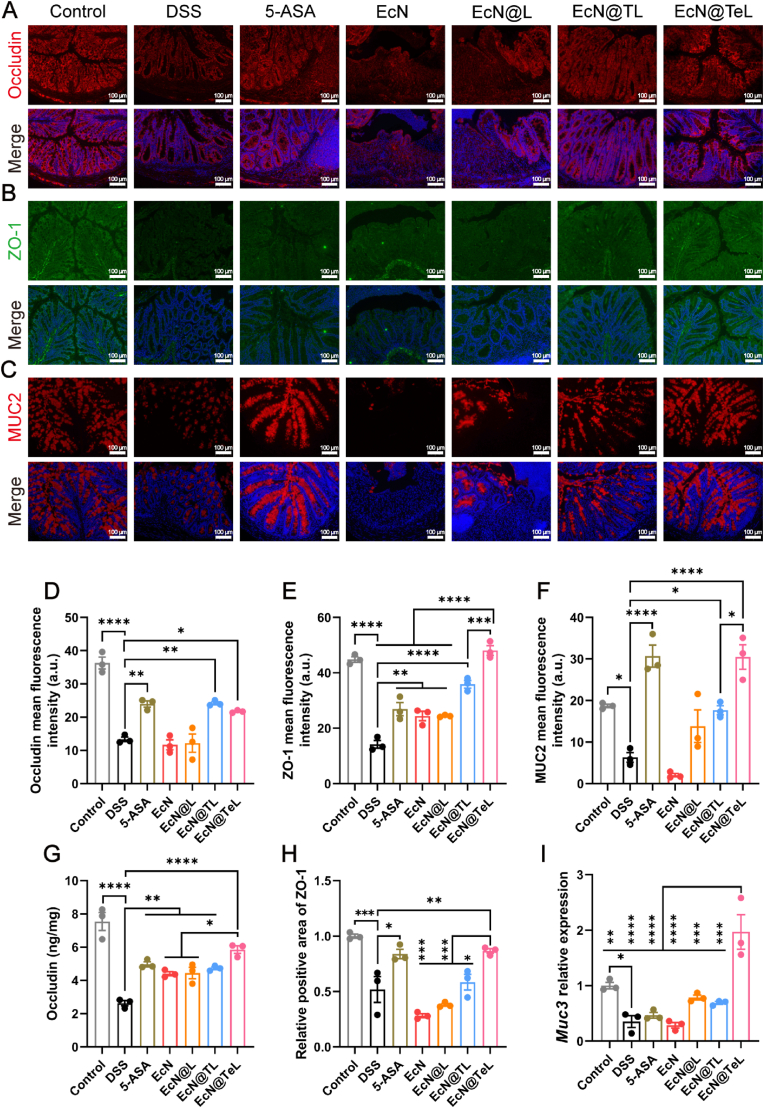
Repair effect of armored probiotics on UC-induced intestinal barrier dysfunction. (A–C) Representative IF staining images of colonic sections showed the levels of Occludin, ZO-1, and MUC2. Scale bars, 100 μm. (D–F) Quantitative analysis based on the mean fluorescence intensity of Occludin, ZO-1, and MUC2 in colonic sections (n = 3). (G) The concentration of Occludin in the colonic tissues (n = 3). (H) Quantitative analysis of immunohistochemically stained sections as shown in Fig. S20 (n = 3). (I) Relative expression of *Muc3* (n = 3). Data were presented as mean ± SEM, statistical analysis was performed using one-way ANOVA, ∗*P* < 0.05, ∗∗*P* < 0.01, ∗∗∗*P* < 0.001, ∗∗∗∗*P* < 0.0001.

According to studies, the collapse of the intestinal barrier is a pivotal feature of UC [55]. Inflammatory factors deplete goblet cells, affect the expression of tight junction proteins, disrupt the junctional complex, and ultimately lead to the declined intestinal permeability and the aberrant transfer of pathogenic microorganisms [56]. The mucus, primarily composed of mucins, is the first line of defense against invasion. Tight junction protein 1 (ZO-1) interacts with other tight junction proteins, such as occludin, to form complexes that mediate cell-cell adhesion and signal transduction, ulteriorly blocking access to pathogens and supporting mucosal immune homeostasis [57]. Their expression levels are integral for maintaining intestinal barrier permeability. As shown in Fig. 5A–C, DSS led to the downregulation of Occludin, ZO-1, and MUC2 levels based on typical fluorescent sections. For Occludin, 5-ASA, EcN@TL, and EcN@TeL significantly restored its fluorescent intensity. Notably, the fluorescence signals of the EcN@TL and EcN@TeL groups on the proximity of mucosa to the lumen were stronger than in the lower part in contact with the lamina propria, implying that EcN preferentially localized to the mucosal surface attributed to the adhesion from the TA coating, and restored the tight junction structure of the intestinal lumen surface, while EcN@TeL possessed the most prominent therapeutic effect on its secretion (Fig. 5D, G). For ZO-1, quantitative analyses histograms of both IF and IHC exhibited a significant inhibitory effect of DSS on its expression, which could be restored by EcN@TeL. In addition, the positive areas of ZO-1 in the colons of the EcN@TeL-treated group were restored to a healthy level (Fig. 5E, H, and Fig. S20). For mucins, fluorescence quantitative analysis and qPCR results denoted that the expression levels of mucins including MUC2 and *Muc3* of mice in EcN@L and EcN@TL groups was close to that of healthy mice, whereas EcN@TeL could prominently enhance the transcription and secretion of mucins. (Fig. 5F, I). Taken together, EcN@TeL not only significantly reversed the colonic damage caused by DSS but also rebuilt the intestinal barrier, which meant that EcN@TeL could facilitate the recovery and secretion of intestinal mucosa, block the contact between pathogenic bacteria and epithelial cells, and promote the adhesion of commensal bacteria to the outer loosely adherent layer of mucus [58,59]. What's more, the levels of ZO-1 and mucin in the EcN@TeL group were considerably higher than that of the EcN@TL group, further confirming the paramount role of antioxidant enzyme loading in the recovery of intestinal barrier dysfunction.

### 3.7 EcN@TeL modulates the gut microbiome disorder caused by DSS

**Fig. 6:**
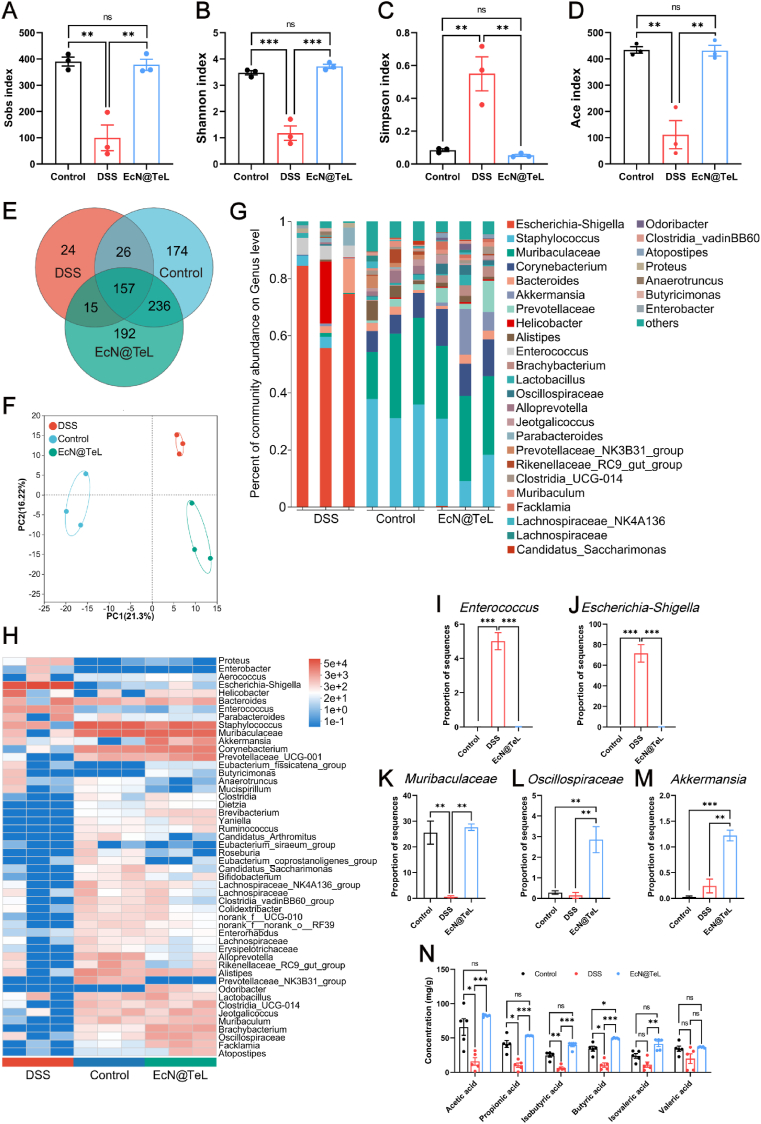
Regulating effect of EcN@TeL on intestinal flora during UC therapy. (A–D) Alpha diversity analysis of microbial communities incorporating the values of Sobs, Shannon, Simpson, and Ace (n = 3). (E) The number of species at the genus level. (F) Genus-level PCA analysis demonstrated the similarities and differences in gut microbiota across groups (n = 3). (G) Relative abundance of gut microbes at genus level. (H) Community heatmap analysis at the genus level. (I–M) Relative abundance of *Enterococcus*, *Escherichia-Shigella*, *Muribaculaceae*, *Oscillospiraceae*, and *Akkermansia* (n = 3). (N) Concentration of SCFAs (n = 5). Data were presented as mean ± SEM, statistical analysis was performed using one-way ANOVA for (A-D), (I-M) and two-way ANOVA for (N), ∗*P* < 0.05, ∗∗*P* < 0.01, ∗∗∗*P* < 0.001, ns, not significant.

There is increasing evidence emphasizing that microbiota dysbiosis is a cause rather than a consequence of UC, which suggests the essential role of gut microbiota in the development of UC [60,61]. Stimulated by the above positive results of *in vivo* assays, we further elucidated the role of EcN@TeL in the regulation of colonic microecology by 16S sequencing analysis. As expected, EcN@TeL significantly restored the alpha diversity indices including community richness (Sobs, Ace, Chao) and diversity (Shannon, Simpson) of colonic microbiota, which had no significance with that of the control group (Fig. 6A–D and Fig. S21). To further explore these findings, we analyzed the compositional structure of species and performed high-dimensional class comparisons *via* LefSe and principal component analysis. As shown in Fig. 6E, the Venn diagram counted the number of common and unique species in three groups, and the lowest number of species were observed in the DSS group, further demonstrating its community homogeneity. Furthermore, gut flora structure, representative branching plot of major bacteria, and PCA scatter plot showed significant differential effects of taxa between control, DSS, and EcN@TeL groups (Fig. S22 and 23, and Fig. 6F).

In addition, the community composition analysis plot at genus level reflected the potential of EcN@TeL in gut flora restoration (Fig. 6G), and the community composition heatmap analysis exhibited a dramatic difference between the DSS and control group, as evidenced by a plunge in conventional species such as *Muribaculaceae* and an abnormal proliferation of conditional pathogenic bacteria such as *Escherichia-Shigella*, *Enterococcus*, and *Proteus* (Fig. 6H). Notably, *Escherichia-Shigella* is significantly enriched in the excreta of patients with UC, which can attach to colonic epithelial cells and elevated the expression of pro-inflammatory cytokines [62]. Its significant expansion is considered one of the characteristics of intestinal ecological dysregulation [63], and the consequent outcome is the loss of beneficial functions like SCFA production [64]. In contrast, EcN@TeL could effectively reverse the above ecological changes to near-normal level. Finally, we visualized and quantified the typical bacterial species, DSS promoted the proliferation of pathogenic bacteria *Enterococcus* and *Escherichia-Shigella* (Fig. 6I–J) and caused a decline in the proportion of normal intestinal genera *Muribaculaceae* (Fig. 6K), whereas EcN@TeL could significantly reverse the above trend and prominently promote the proliferation of probiotics *Oscillospiraceae* and *Akkermansia* (Fig. 6L–M). *Oscillospiraceae* is a butyrate producer [65], the abundance of which is positively correlated with microbial diversity and high-density lipoprotein, and it has been characterized as a candidate for the next-generation probiotics [66,67]. *Akkermansia*, one of the well-known probiotics, has been extensively documented to exert numerous beneficial effects including anti-inflammatory, maintaining the integrity of the epithelial cells and mucus layer, and even fighting obesity, diabetes, and aging [[68], [69], [70]]. In addition, compared to the control group, the increase in *Akkermansia* abundance in the DSS group may be due to the following reasons: On the one hand, IBD leads to a reduced source of nutrients for gut flora, with an impaired intestinal barrier and more exposure of the mucus components, whereas *Akkermansia* can utilize mucin as the only source of carbon and nitrogen to proliferate and remain relatively stable in the intestinal tract. On the other hand, *Akkermansia* may be “recruited” by the body in response to intestinal inflammation due to its multiple salutary functions [71]. Short-chain fatty acids can be secreted by probiotics after the fermentation of carbohydrates, which are closely related to the microbial community homeostasis. Encouraged by the salutary regulation of EcN@TeL to beneficial bacteria, we further monitored the SCFAs content in the gut. As shown in Fig. 6N, the concentrations of SCFAs in feces from inflamed intestines were remarkably lower than those of the control group, whereas EcN@TeL could revert the SCFAs to normal level, in which the concentration of butyric acid even exceeded that of the control group strikingly. The recovery of SCFAs might be a result of the combined effect of improved intestinal flora and EcN@TeL, demonstrating that EcN@TeL could directly or indirectly expedite the SCFAs generation. Taken together, EcN@TeL may play a therapeutic role in UC by regulating the gut microbial ecology, optimizing the intestinal flora, and regaining the SCFAs secretion.

### 3.8 Prophylactic effect of EcN@TeL on ulcerative colitis

**Fig. 7:**
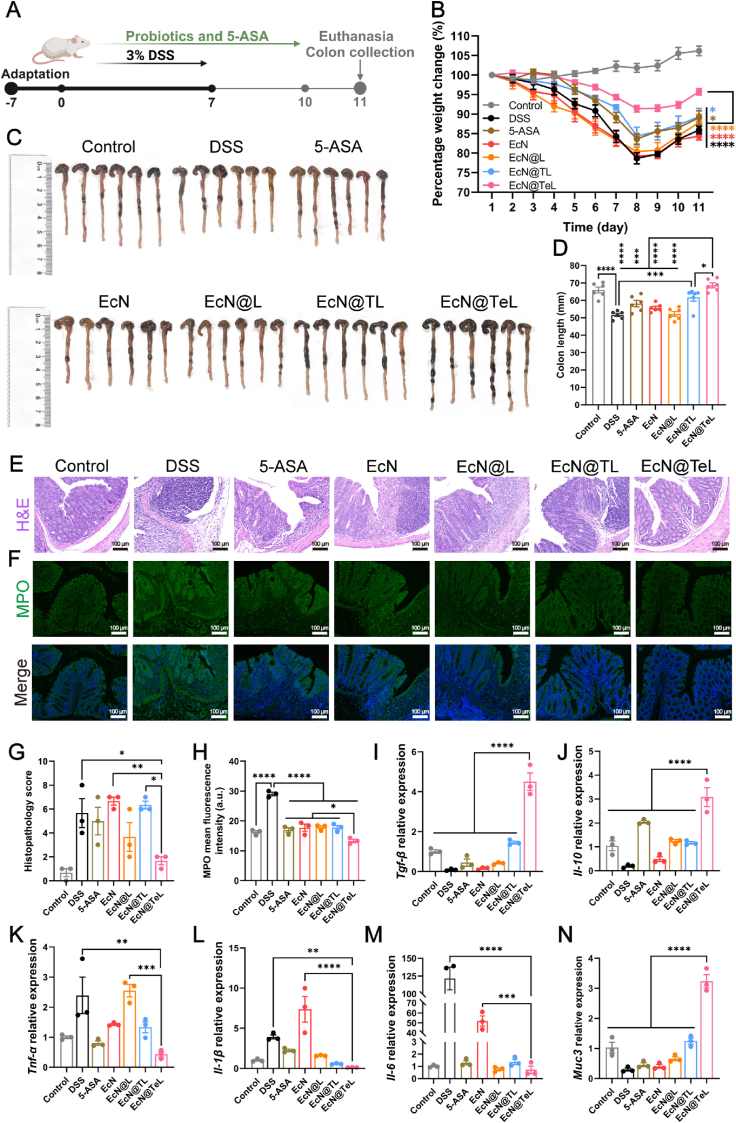
Prophylactic effect of EcN@TeL on ulcerative colitis. (A) Schematic diagram of the experimental procedure showed the treatment of DSS-endured mice. (B) Percentage changes in body weight of DSS-endured mice with different treatments for 10 days (n = 6). (C) Colon images of UC mice. (D) Quantitative analysis of colonic length (n = 6). (E–F) Representative H&E− and IF-stained colonic section images of mice with different treatments. Scale bars, 100 μm. (G) Quantitative analysis of pathology scores according to Table S2 (n = 3). (H) Quantitative analysis of mean MPO fluorescence intensity (n = 3). (I–N) Relative mRNA expression of UC-associated factors including *Tgf-β*, *Il-10*, *Tnf-α*, *Il-1β*, *Il-6*, and *Muc3* in colon tissues of DSS-beared mice (n = 3). Data were presented as mean ± SEM, statistical analysis was performed using one-way ANOVA for (D), (G–N), and two-way ANOVA for (B), ∗*P* < 0.05, ∗∗*P* < 0.01, ∗∗∗*P* < 0.001, ∗∗∗∗*P* < 0.0001.

To further verify the diversification of therapeutic effects of EcN@TeL on UC, DSS was added to the drinking water with a mass fraction of 3 % of all groups except the control group for 7 days to induce acute colitis, and the mice were administered saline, respective probiotic and 5-ASA (EcN, 5 × 10^7^ CFU/d; 5-ASA, 250 mg/kg/d) *via* gavage for 10 days to simulate preventive medication (Fig. 7A). As shown in Fig. 7B and Fig. S24, except for the 5-ASA and EcN@TeL group, all other treated groups showed no significant difference in weight loss and DAI compared to the DSS group. In addition, EcN@TeL unsurprisingly restored the pathological colons to a healthy level, with a similarity in colon length compared with the control group (Fig. 7C–D). It is noteworthy that the colons in the EcN@TeL group were significantly longer than in the EcN@TL group, manifesting the advantageous role of enzyme embedding in inflamed colon recovery. Furthermore, EcN@TeL could cure colonic inflammatory symptoms such as crypt vanishing, elcosis, and inflammatory infiltration, resulting in an obvious decrease in the pathological score, and the pathological score based on H&E staining of the EcN@TeL group was evidently lower than that of the EcN@TL group (Fig. 7E, G). In addition, EcN@TeL notably reduced the DSS-induced spleen index elevation, while the EcN@TL group showed no significant difference from the DSS group (Fig. S25), certifying the enormous potential of enzyme loading in coordinating immune cells and controlling inflammatory responses during UC therapy. What's more, according to the IF-stained sections of colonic tissues, EcN@TeL could normalize the MPO expression, evidencing that EcN@TeL could effectively prevent the impending oxidative damage attributed to colitis (Fig. 7F, H). Further transcriptional assay results demonstrated that EcN@TeL could promote the expression of anti-inflammatory factors like *Tgf-β* and *Il-10* (Fig. 7I–J), reduce the expression of pro-inflammatory factors such as *Tnf-α*, *Il-1β* and *Il-6* (Fig. 7K–M), and significantly recover the mRNA expression of *Muc3* (Fig. 7N). These results highlighted that EcN@TeL demonstrated remarkably prophylactic efficacy against DSS-induced UC.

Encouraged by the satisfactory performance of the preventative effect of EcN@TeL, we further investigated whether it could also modulate gut microbiota. As shown in Fig. S26A–C, EcN@TeL could reconstruct the abundance and diversity of the gut microbiome, and positively coordinate the differential effects and phylum-level community composition (Fig. S26D–F). Finally, we performed visual and quantitative analysis on the bacterial species with significant differences and found that the abundance of the core gut microbiota, *Bacteroidota*, was effectively restored after the EcN@TeL treatment. *Bacteroidota*, as a dominant phylum, can metabolize carbohydrates, participate in metabolic activity and immune regulation, and contribute to the reduction of inflammation and obesity [72]. At the genus level, compared to DSS group, the analysis showed a noteworthy increase in the abundance of *Parabacteroides goldsteinii*, *Enterococcus faecalis*, and *Romboutsia* in the EcN@TeL group (Fig. S26H–J). Among them, *Parabacteroides goldsteinii* has been recognized as a potential next-generation probiotic due to its capacity to improve obesity, reduce intestinal barrier damage, prevent intestinal injury, and ameliorate intestinal inflammation in mice [73,74]. *Enterococcus faecalis*, as a commensal bacterium in the gut microbiota, can not only form biofilms on the surface of intestinal epithelial cells to protect the gut barrier but also produce bacteriocins to inhibit pathogen proliferation, which makes it integral in improving microbiome balance [75]. *Romboutsia* is a recently discovered genus, typically associated with the health status of patients, its reduction may be linked to colorectal carcinogenesis, hypertension, and mental health disorders [76]. In conclusion, the above findings emphasized the potential of EcN@TeL to prevent UC through immune modulation, gut recovery, and microbiome adjustment, thereby exerting a therapeutic effect.

## 4 Discussion

In recent years, gut-targeted probiotic delivery systems have received increasing attention for their adjunctive augmentation of therapeutic effects on UC, demonstrating numerous possibilities and prospects in the treatment of intestinal diseases [[77], [78], [79]]. However, previous attempts have mainly focused on utilizing nanocapsules to deliver probiotics or functional drugs to cure UC by alleviating microbiota imbalance. Although these endeavors are promising and have clinical application value to some extent, the dynamic problems such as the short transport time of probiotics and the high concentration of ROS in the pathological gut have not been effectively solved [80]. To fill these gaps, the present study utilized the adsorptive property of TA and the pH-responsiveness of L100 to design an enzyme-loadable multiplexed encapsulation-release strategy and interpreted the functional regulation of probiotics in the system. Compared to bare EcN, the functional coatings significantly enhance the probiotics’ resistance to harsh conditions like UV light, H_2_O_2_, antibiotics, and SGF by millions of times. They also promoted the release and adhesion of probiotics in the intestinal tract, EcN@T showed over a 6-fold increase *in vitro* adhesion capacity compared to native EcN. The TA-L100 coating heightened the colonization of EcN in the intestine for up to 72 h, and helped EcN to maintain a colonization advantage in the proximal intestine, where bare EcN lost their effectiveness. The above results demonstrate that this strategy can increase the EcN number exposed in intestines and prolong the transit time of it.

In addition, the goals of clinical drugs are mainly focused on immune modulation and inflammation remission, while ROS clearance and histological improvement are scant concern [81,82]. The overproduction of ROS is a common feature of all inflammatory diseases, with the ROS concentration in inflammatory areas being 10–100 times higher than in healthy tissues [83,84], which can cause oxidative stress in intestinal epithelial cells and gut flora, preventing beneficial bacteria from colonizing the inflamed sites and further contributing to dysfunction of the intestinal environment [85]. In our work, CAT and SOD were embedded *in situ* in the functional shell to enhance its antioxidant property. EcN@Te could productively scavenge ROS from the surroundings, inhibit MPO expression, and reduce ROS accumulation in the colons, thereby alleviating oxidative stress in intestinal microbiota and inflammatory tissues.

The imbalance of intestinal flora, mainly characterized by colossal changes in the ratio of normal flora species, can influence the occurrence and development of UC through various pathways including enteral microbial factors, aberrant immune responses, and a compromised intestinal mucosal barrier [86,87]. Our multiplexed encapsulation-release strategy enabled the targeted release of EcN@Te into the gut, especially in inflammatory areas. This was due to the pH-triggered-solubilization property of L100 and the interaction between the positively charged proteins overexpressed in inflamed regions and the negatively charged surface of EcN@T. The antioxidant enzymes within the shell could scavenge excessive ROS, and the TA shell could promote the adhesion of probiotics within the colon. In addition, the iron ions in Fe^3+^-TA coatings could be absorbed by probiotics and participate in various reactions including catalytic reactions, electron transfer, and DNA synthesis [88]. Tannic acid could be broken down and metabolized by microflora, thereby regulating the intestinal microenvironment. Afterward, probiotics were activated and resumed growth after a short delay in activity. They repaired the intestinal barrier, adjusted the ratio of pathogenic bacteria to probiotics, and regulated the intestinal microenvironment, thus realizing a multi-pronged approach to the management of UC.

## 5 Conclusion

In summary, we constructed an enzyme-loadable multifaceted regulatory platform for targeted delivery of probiotics and antioxidant enzymes to colon without threat from digestive fluid. The antioxidant system consisted of CAT, SOD, and TA shells that rapidly scavenged ROS from the inflammatory microenvironment. In addition, the retention time of EcN was prolonged benefiting from the inherent adhesion capacity of the TA shell. Mitigatory inflammation enhanced bacterial viability to rapidly reshape the intestinal barrier and gut microbiota, thereby significantly improving the efficacy of probiotics on the prevention and alleviation of acute UC symptoms in mice and achieving multidimensional regulation of intestinal diseases. And there is no observed detrimental event in mice following repeated EcN@TeL treatments. In addition, the resistance of the strategy to antibiotics makes it possible to combine probiotic therapy with antibiotics, bringing further benefits for personalized treatment and precision medicine. These results emphasize the promising potential of EcN@TeL as a therapeutic and support further clinical evaluation of various types of IBD. However, because of the significant differences in physiological structures and pathological responses between mice and humans, this study awaits further large animal experiments to elucidate the underlying rationale.

## CRediT authorship contribution statement

**Zhishu Li:** Writing – review & editing, Writing – original draft, Visualization, Investigation. **Xinlin Wei:** Investigation, Methodology, Visualization. **Wenting Chen:** Visualization, Methodology, Investigation. **Xuelian Qiu:** Visualization, Methodology, Investigation. **Jieyan Shi:** Visualization, Methodology, Investigation. **Yu Li:** Visualization, Methodology, Investigation. **Zhixuan Wang:** Visualization, Methodology, Investigation. **Xiaolin Chen:** Visualization, Methodology, Investigation. **Yuepeng Wang:** Visualization, Methodology, Investigation. **Lizeng Cheng:** Visualization, Methodology, Investigation. **Bo Teng:** Visualization, Methodology, Investigation. **Harold Corke:** Visualization, Methodology, Investigation. **Bo-Bo Zhang:** Writing – review & editing, Visualization, Supervision, Investigation, Conceptualization. **Qiongqiong Yang:** Writing – review & editing, Writing – original draft, Visualization, Supervision, Investigation, Conceptualization.

## Ethics approval and consent to participate

All animal experiments involved were reviewed and approved by the Laboratory Animal Ethics Committee of Shantou University Medical College, and the relevant animal care guidelines were followed (SUMC2023-480). All authors had given approval to the final version of the manuscript and agreed to submit.

## Funding

This work was supported by the Special Funding Program for Guangdong Science and Technology Innovation Strategic Project [STKJ202209022]; the 10.13039/501100001809National Natural Science Foundation of China [32302280, 32072184, and 32472313]; and the Research Start-up Foundation of 10.13039/100009047Shantou University [NTF22003].

## Declaration of competing interest

The authors declare that they have no known competing financial interests or personal relationships that could have appeared to influence the work reported in this paper.

## Data Availability

Data will be made available on request.
