# Supplementary material for: Decorating probiotics with a triggerable and catalytic shell for synergistically enhanced colitis biotherapy
Source: Mater Today Bio. 2025 May 12;32:101861. doi: 10.1016/j.mtbio.2025.101861 (PMC12144505; doi:10.1016/j.mtbio.2025.101861)
Supplement: Multimedia component 1 [file mmc1.doc]

**Supplementary Materials**

**Decorating probiotics with a triggerable and catalytic shell for synergistically enhanced colitis biotherapy**

Zhishu Li ^a^, Xinlin Wei ^f^, Wenting Chen ^a^, Xuelian Qiu ^b^, Jieyan Shi ^a^, Yu Li ^a^, Zhixuan Wang ^a^, Xiaolin Chen ^a^, Yuepeng Wang ^a^, Lizeng Cheng ^f^, Bo Teng ^a^, Harold Corke ^g,h^, Bo-Bo Zhang ^a,c,d,e,**^, Qiongqiong Yang ^a,c,d,e,*^

^a^ *Department of Biology, College of Science, Shantou University, Shantou 515063, Guangdong, China*

^b^ *Guangdong Yichao Biological Co., Ltd., Shantou 515064, Guangdong, China*

^c^ *Guangdong Provincial Key Laboratory of Marine Biotechnology, Institute of Marine Sciences, Shantou University, Shantou, 515063, China*

^d^ *Shantou Key Laboratory of Marine Microbial Resources and Interactions with Environment, Shantou University, Shantou, 515063, China*

^e^ *Guangdong Branch of State Key Laboratory for Esophageal Cancer Prevention and Treatment, Shantou 515063, Guangdong, China*

^f^ *School of Agriculture and Biology, Shanghai Jiao Tong University, Shanghai 200240, China*

^g^ *Biotechnology and Food Engineering Program, Guangdong Technion-Israel Institute of Technology, Shantou 515063, Guangdong, China*

^h^ *Faculty of Biotechnology and Food Engineering, Technion-Israel Institute of Technology, Haifa 320003, Israel*

*Corresponding authors.

**Corresponding author.

*E-mail addresses*: qiongqiongyang@stu.edu.cn (Q. Yang), bbzhang@stu.edu.cn (B. -B. Zhang).


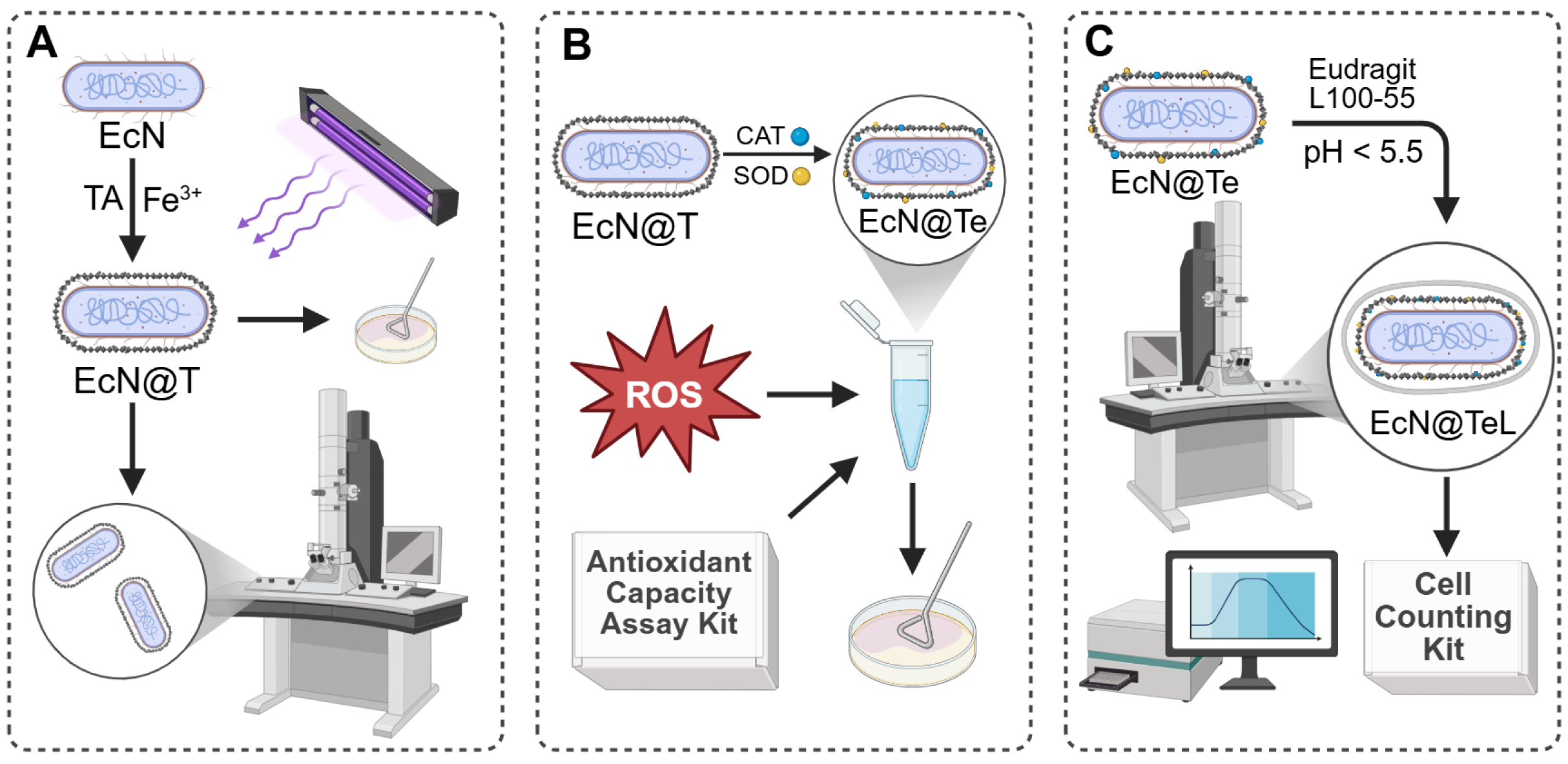


**Fig. S1.** Schematic diagram of the preparation, characterization, and functional verification of (A) EcN@T, (B) EcN@Te, and (C) EcN@TeL.

**
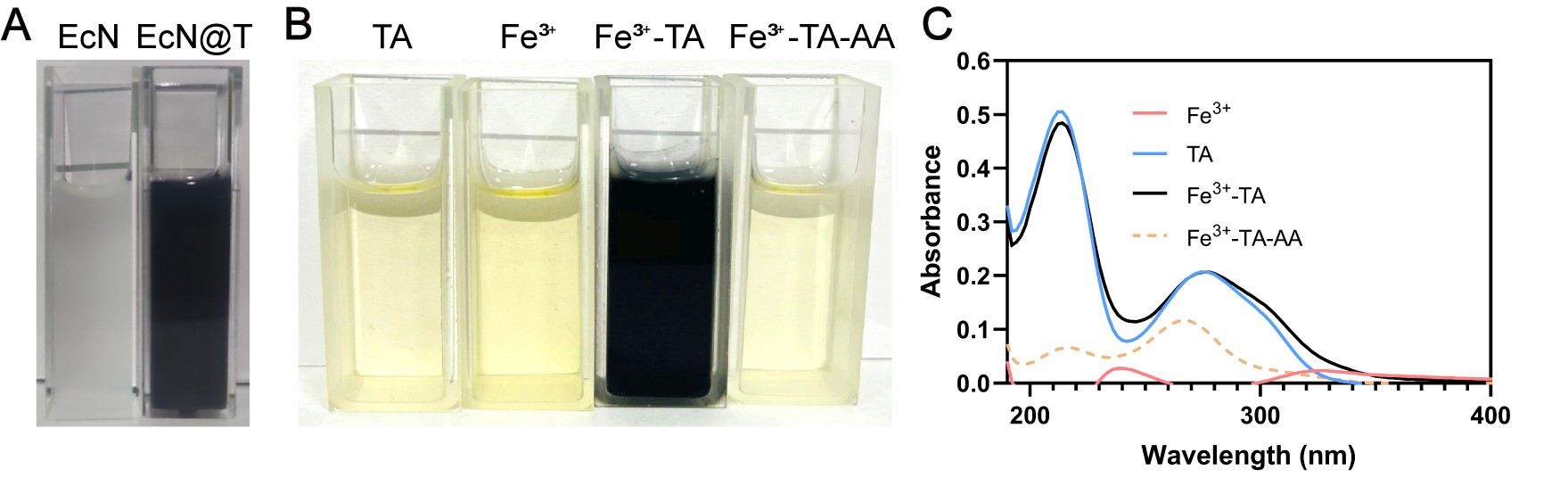
**

**Fig. S2.** (A) Photographs of EcN and EcN@T. (B) Photographs of TA, Fe^3+^, Fe^3+^-TA, and Fe^3+^-TA-AA solutions without EcN. (C) UV spectrums of TA, Fe^3+^, Fe^3+^-TA, and Fe^3+^-TA-AA solutions without EcN.


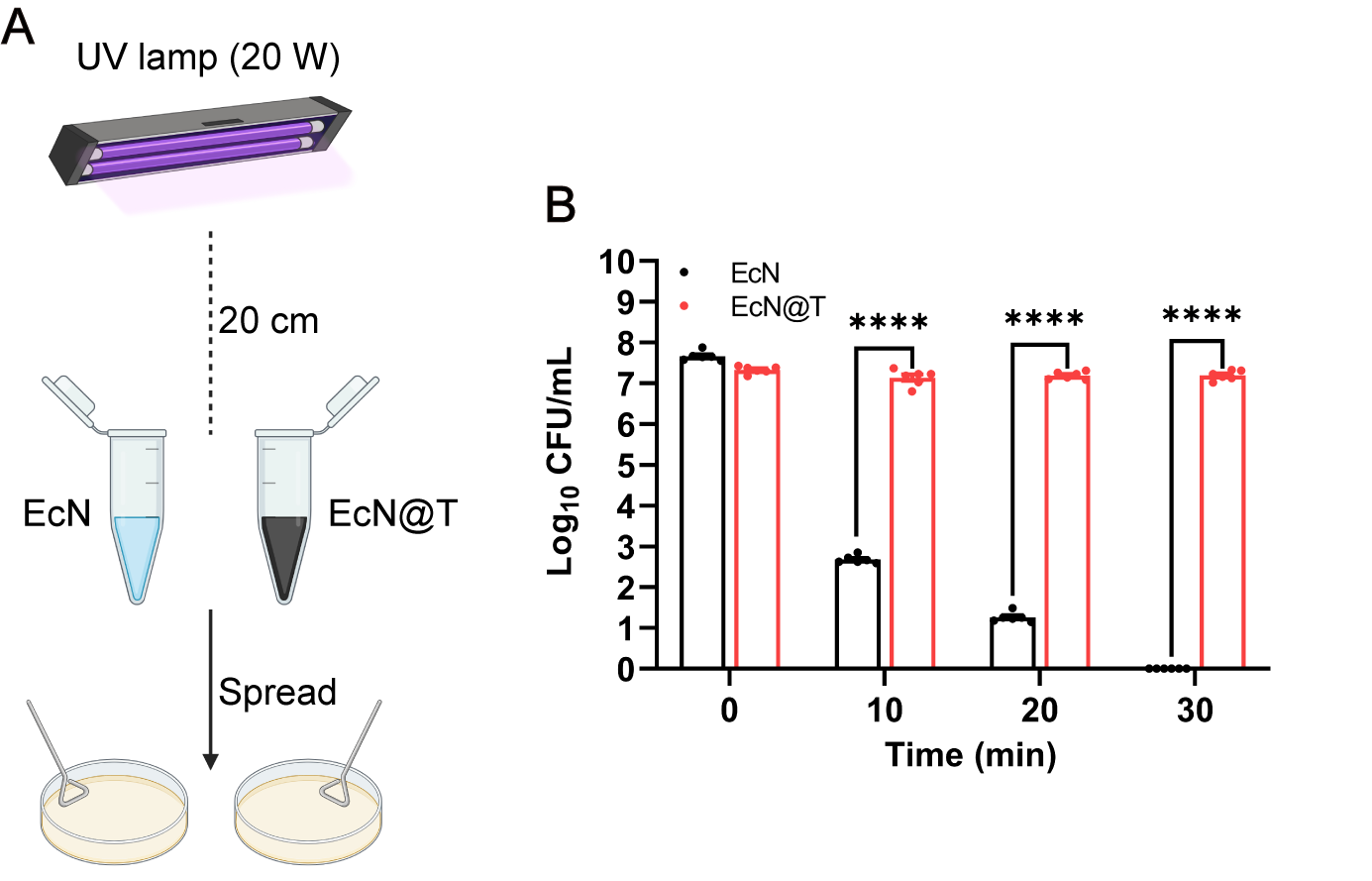


**Fig. S****3.** (A) Schematic diagram of the resistance of EcN to ultraviolet irradiation (20 W, 20 cm). (B) Bacterial counts after the treatment with UV light (n = 6). Data were presented as mean ± SEM, statistical analysis was performed using two-way analysis of variance (ANOVA), *****P* < 0.0001.


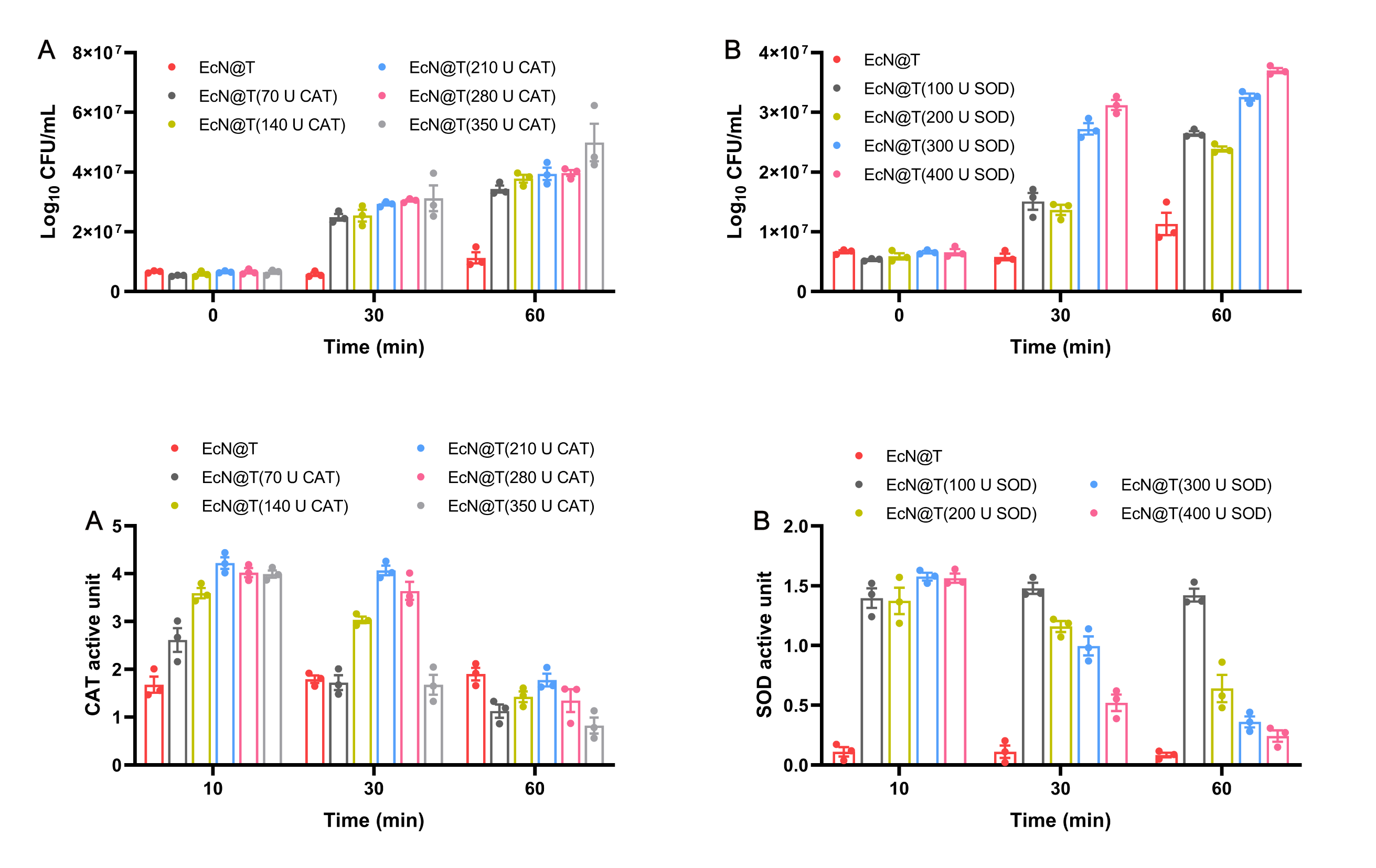


**Fig. S4.** Bacterial counts of EcN@T incubated with different amounts of (A) CAT and (B) SOD for different times.


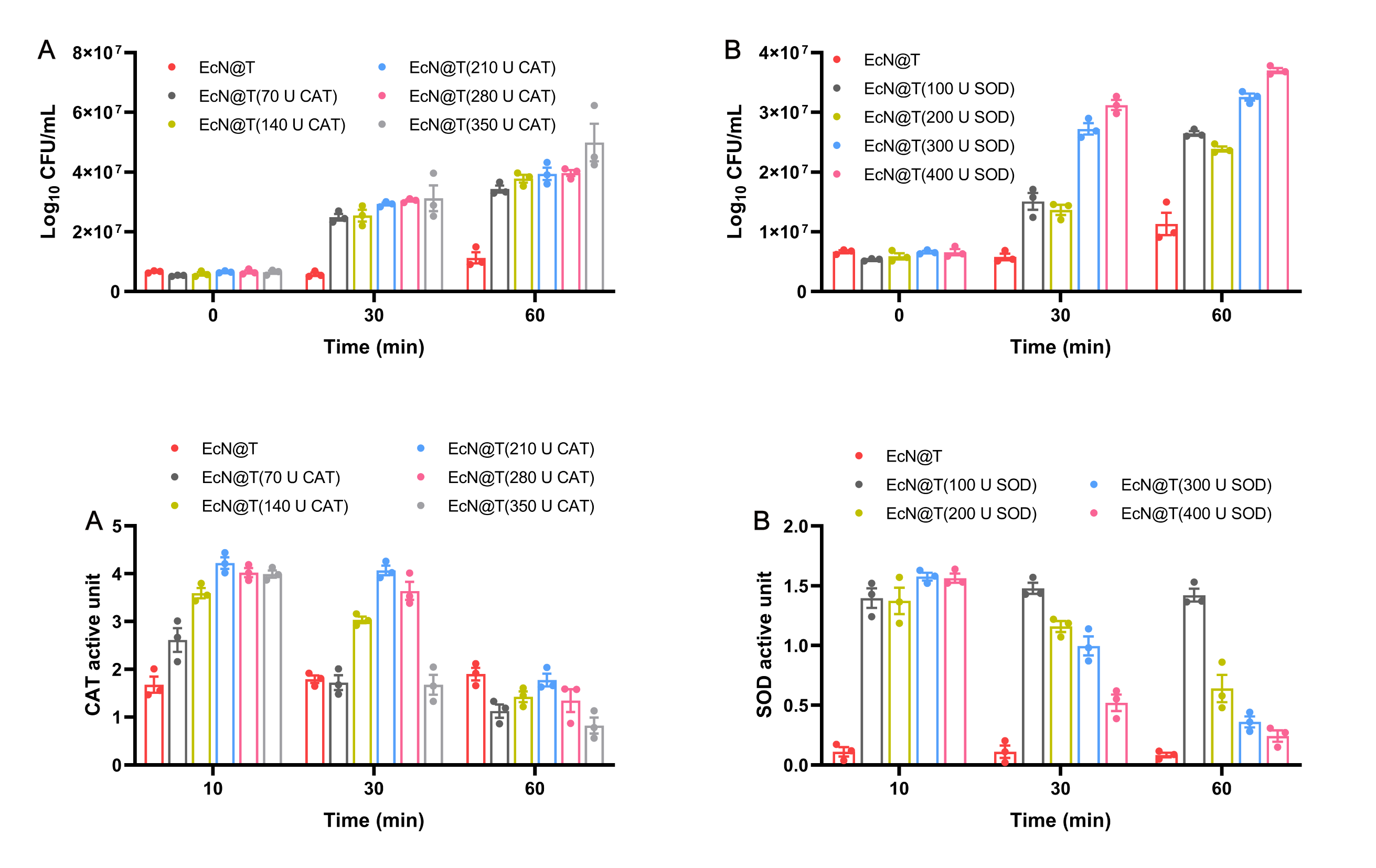


**Fig. S5.** Enzyme active assays of EcN@T incubated with different amounts of (A) CAT and (B) SOD for different times.


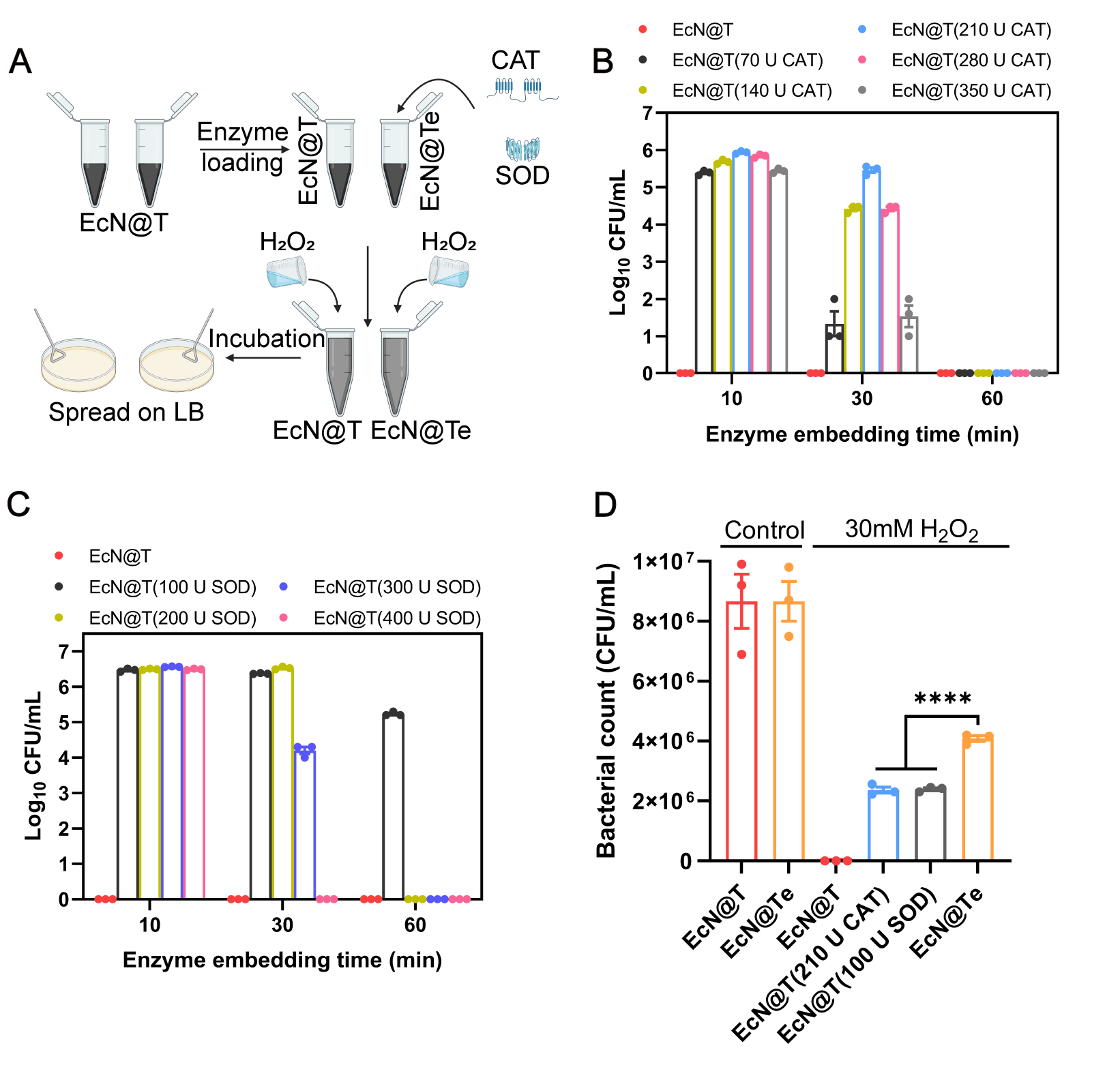


**Fig. S****6.** The loading effect of CAT and SOD was verified using H_2_O_2_. (A) Schematic diagram of enzyme loading and antioxidant procedures. (B) Bacterial counts of EcN@T loaded with varying amounts of CAT at different time points following 60-minute treatment with H_2_O_2_ (15 mM) (n = 3). (C) Bacterial counts of EcN@T loaded with varying amounts of SOD at different time points following 60-minute treatment with H_2_O_2_ (15 mM) (n = 3). (D) Survival of EcN@T loaded with CAT and/or SOD for 10 min after the treatment with H_2_O_2_ (15 mM) for 60 min (n = 3). Data were presented as mean ± SEM, statistical analysis was performed using one-way ANOVA for (D), and two-way ANOVA for (B-C), **P* < 0.05, ****P* < 0.001.


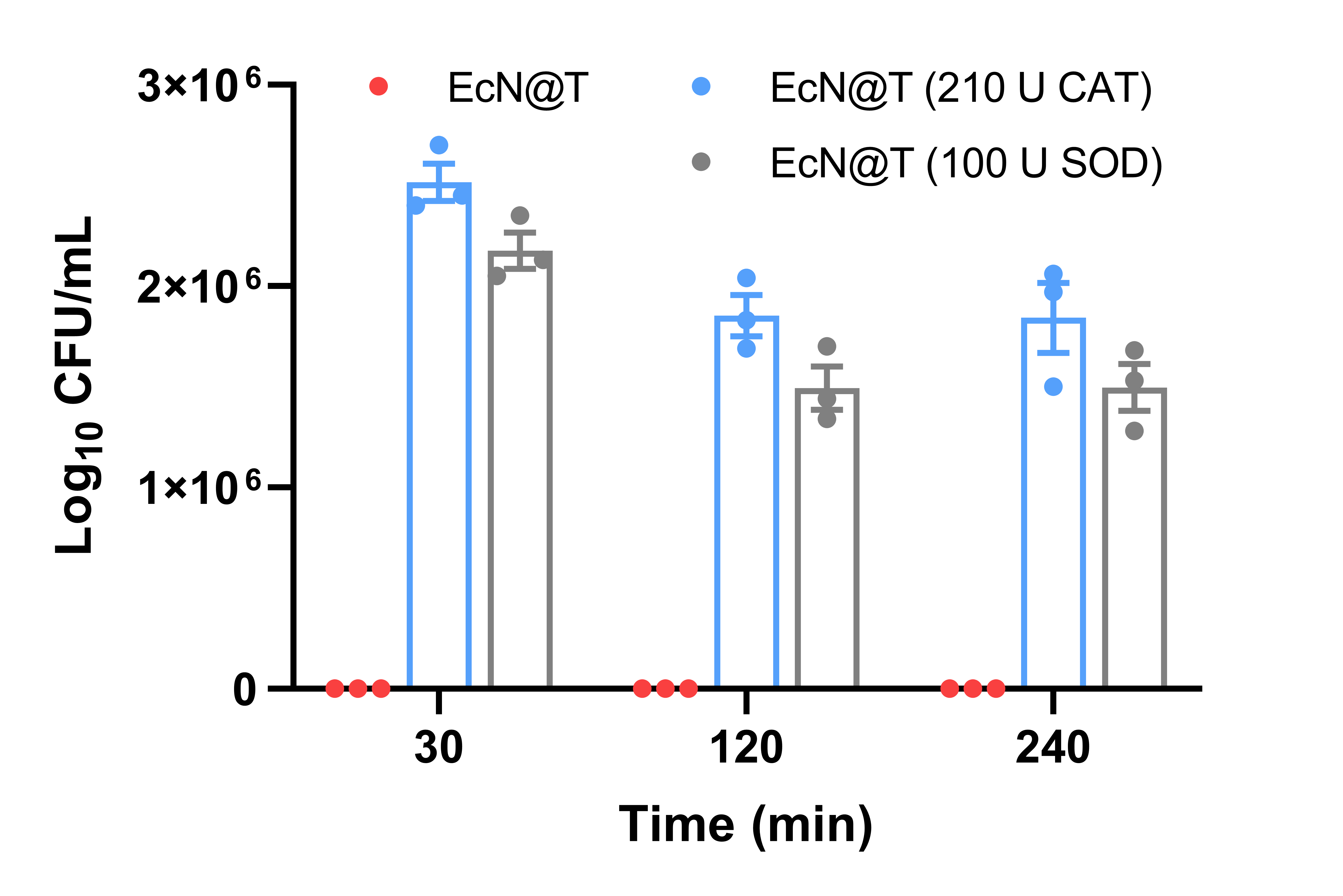


**Fig. S7.** Bacterial counts of EcN@T loaded with CAT or SOD after storage for different periods of time and H_2_O_2_ treatment.


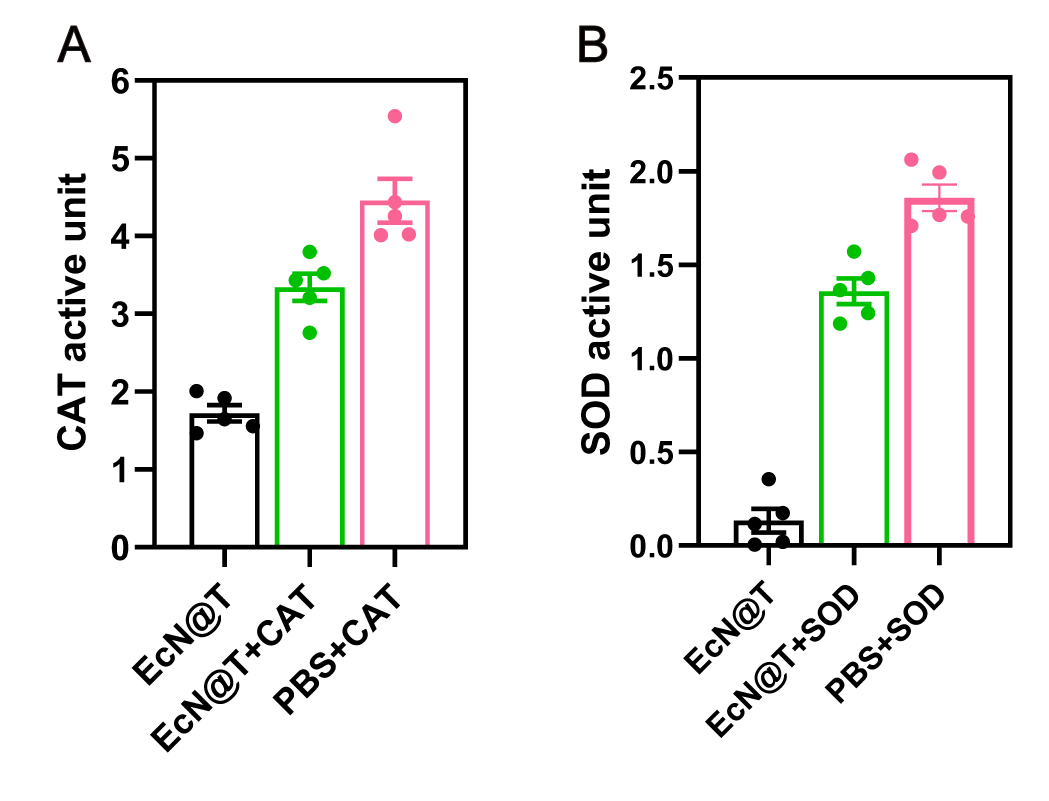


**Fig. S8.** (A) CAT active assay (n = 5). (B) SOD active assay (n = 5). EcN@T was used as a control and enzyme-contained PBS was used as a positive control.


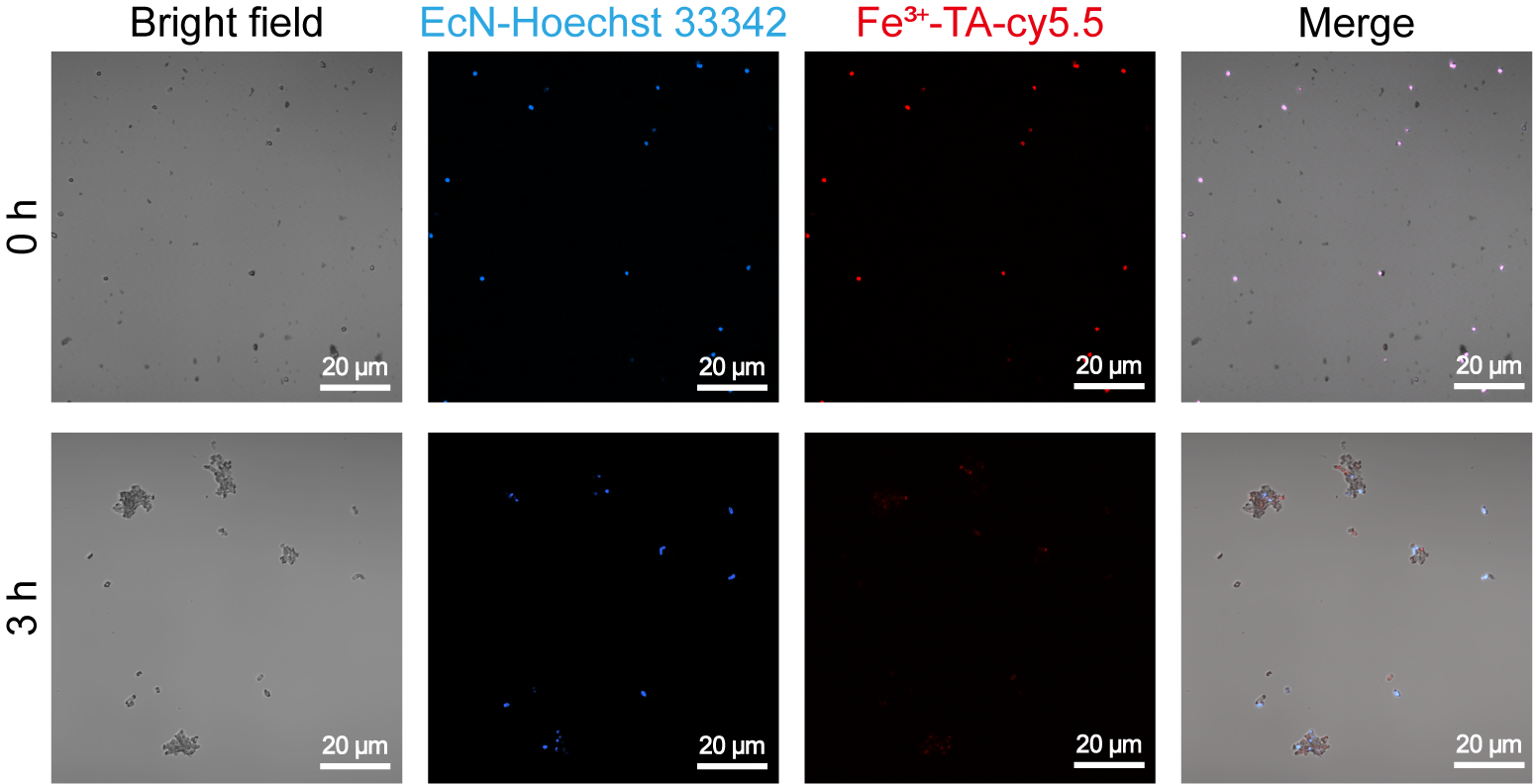


**Fig. S9.** Representative LCSM images of EcN@T with or without incubation in PBS for 3 h. Scale bars, 20 μm.


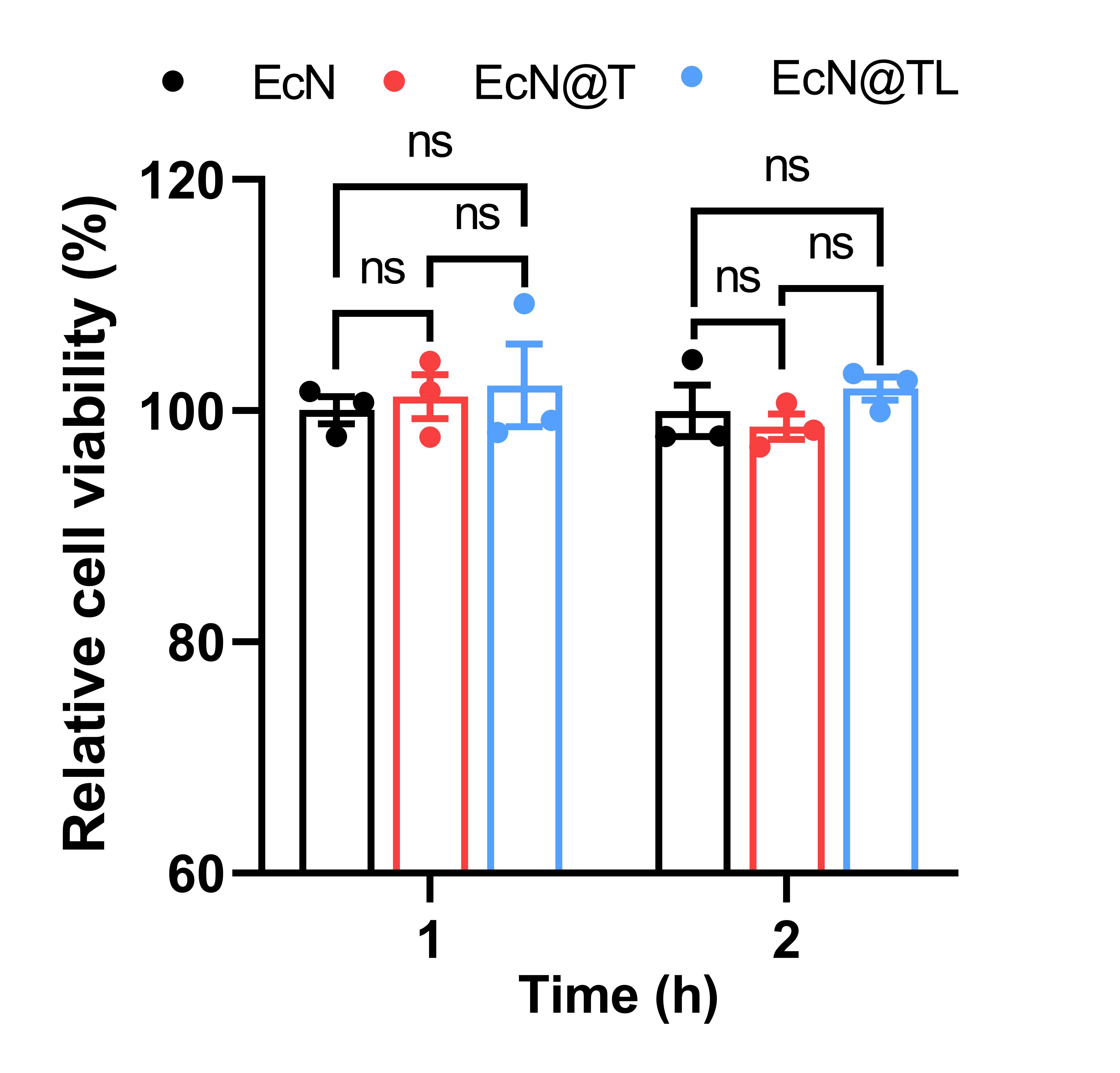


**Fig. S10.** Cell viability assays of EcN, EcN@T, and EcN@TL after 2 h of encapsulation. (n = 3). Data were presented as mean ± SEM, statistical analysis was performed using one-way ANOVA, ns, not significant.


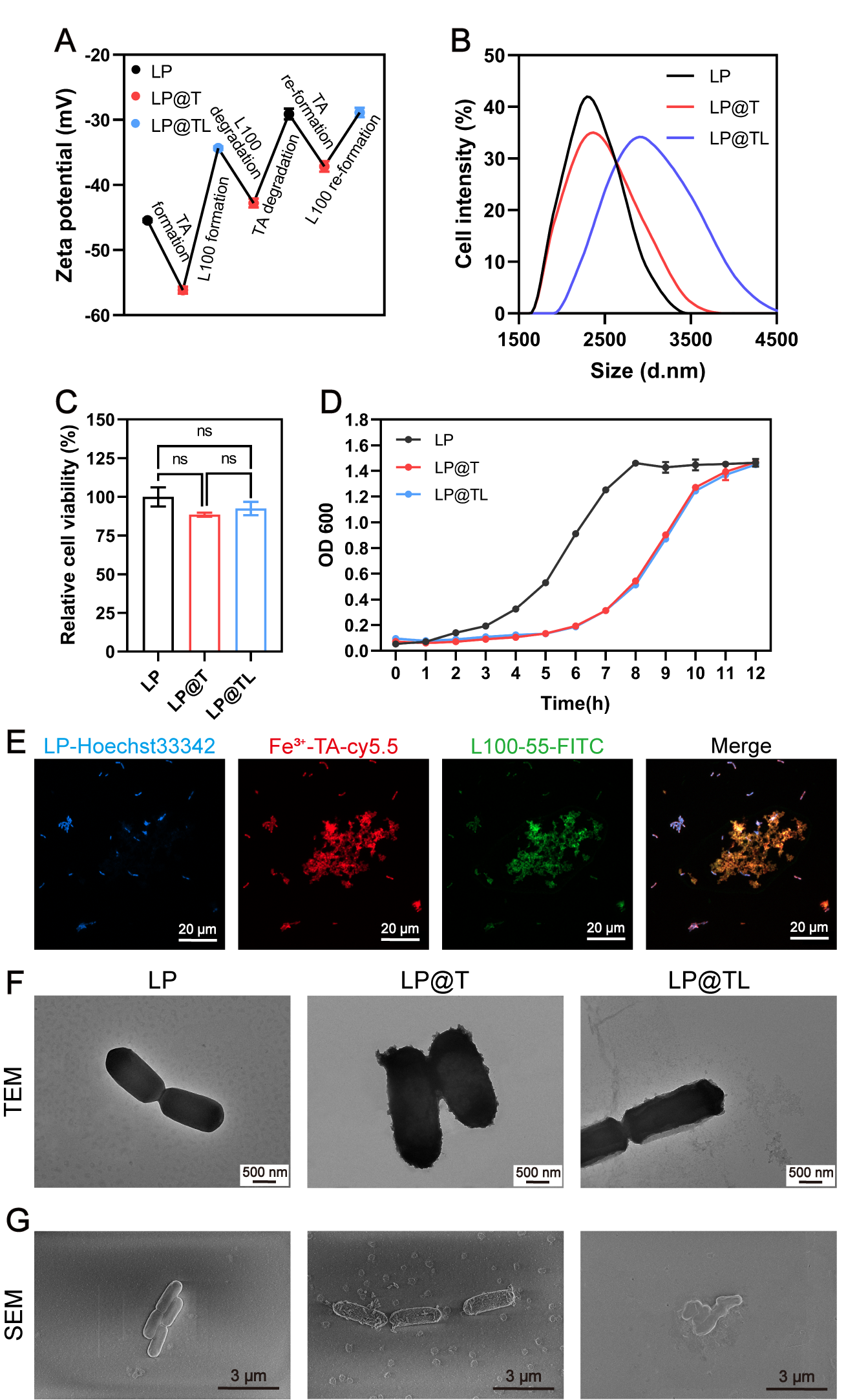


**Fig. S11.** Preparation and characterization of LP, LP@T, and LP@TL. (A) Changes in zeta potential during the encapsulation and degradation of TA and L100 (n = 6). (B) Cell size of LP, LP@T, and LP@TL. (C) Cell viability assays of LP, LP@T, and LP@TL (n = 3). (D) Growth curves of LP, LP@T, and LP@TL (n = 3). (E) Typical LCSM images of LP@TL. Scale bars, 20 μm. (F-G) Representative TEM and SEM images of LP, LP@T, and LP@TL. Scale bars, 500 nm and 3 μm, respectively. Data were presented as mean ± SEM, statistical analysis was performed using one-way ANOVA, ns, not significant.


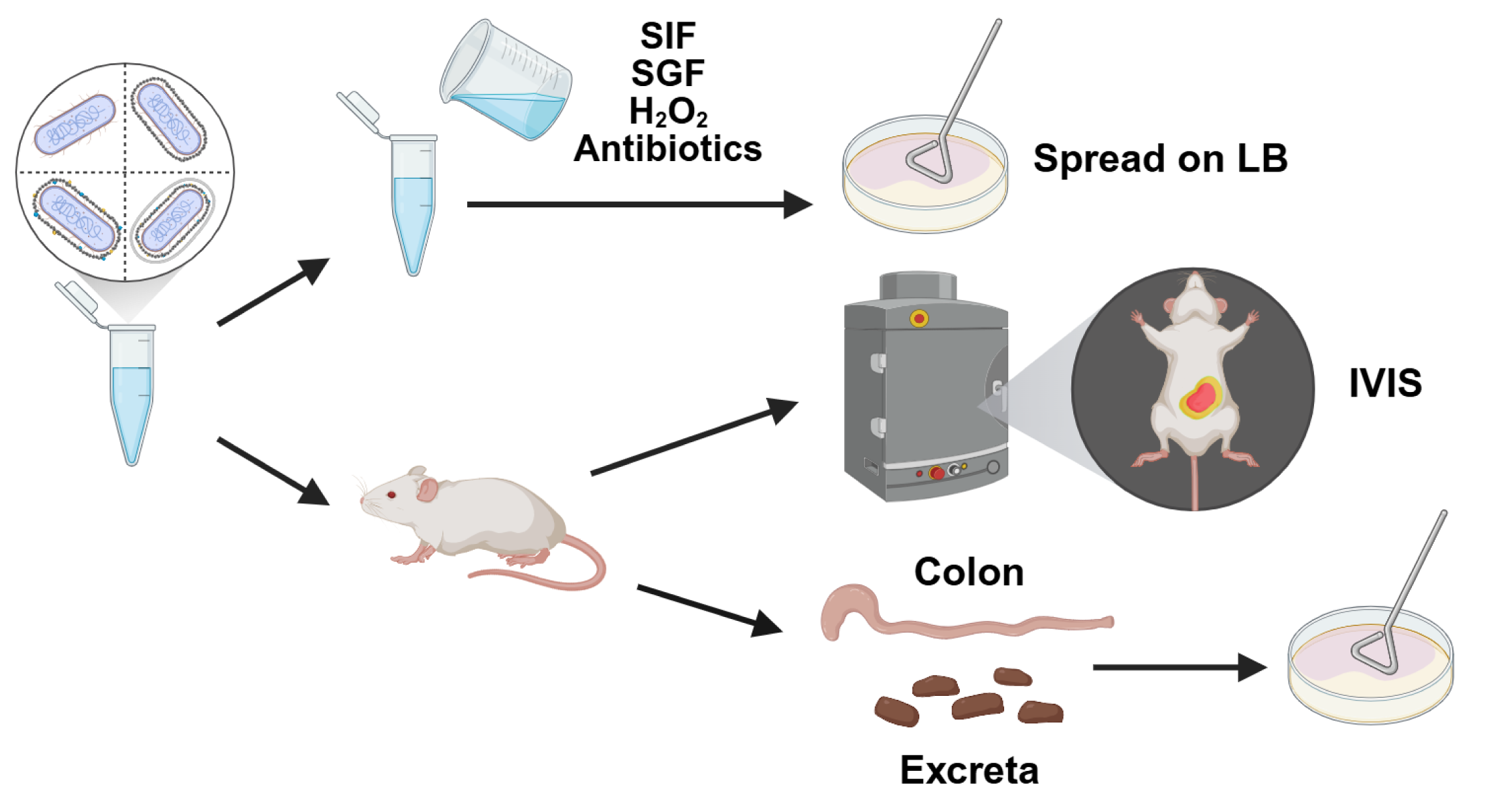


**Fig. S12.** Schematic diagram of the bioavailability assays of enveloped EcN *in vitro* and *in vivo*.


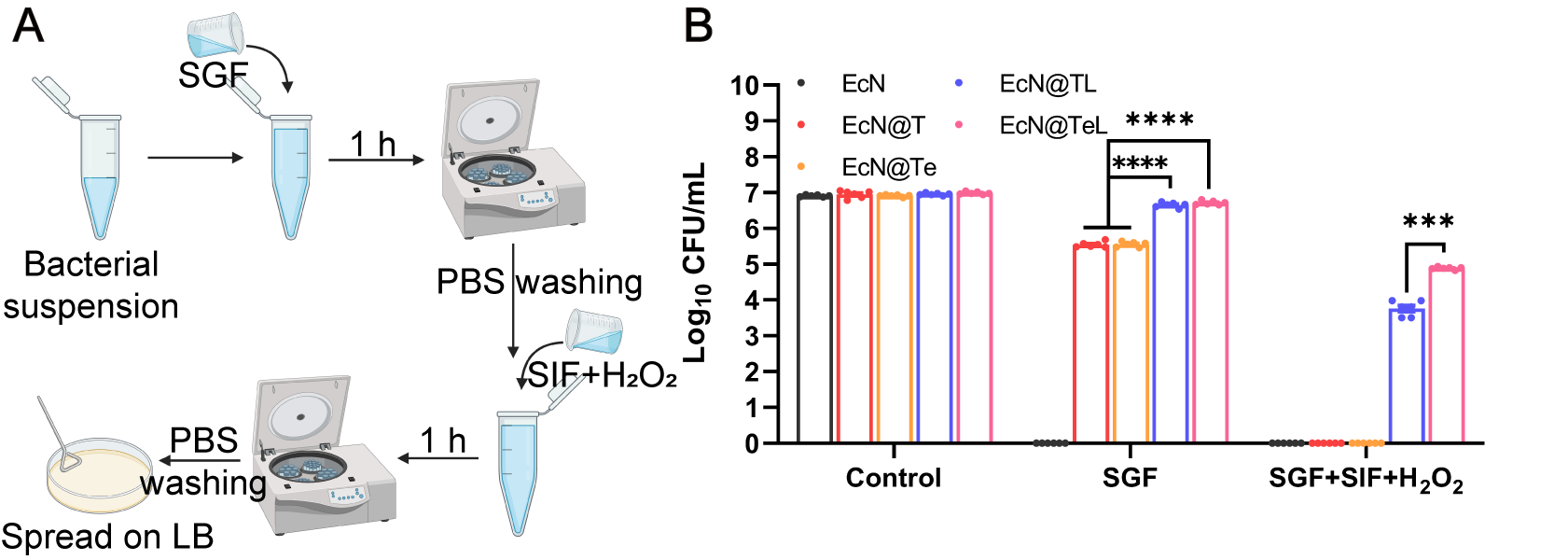


**Fig. S13.** (A) Diagram of the resistance of probiotics in different treatment groups against the digestion attributed to simulating inflammatory gastrointestinal fluid. (B) Plate colony counts of EcN, EcN@T, EcN@Te, EcN@TL and EcN@TeL after digestion (n = 6). Data were presented as mean ± SEM, statistical analysis was performed two-way ANOVA, ****P* < 0.001, *****P* < 0.0001.


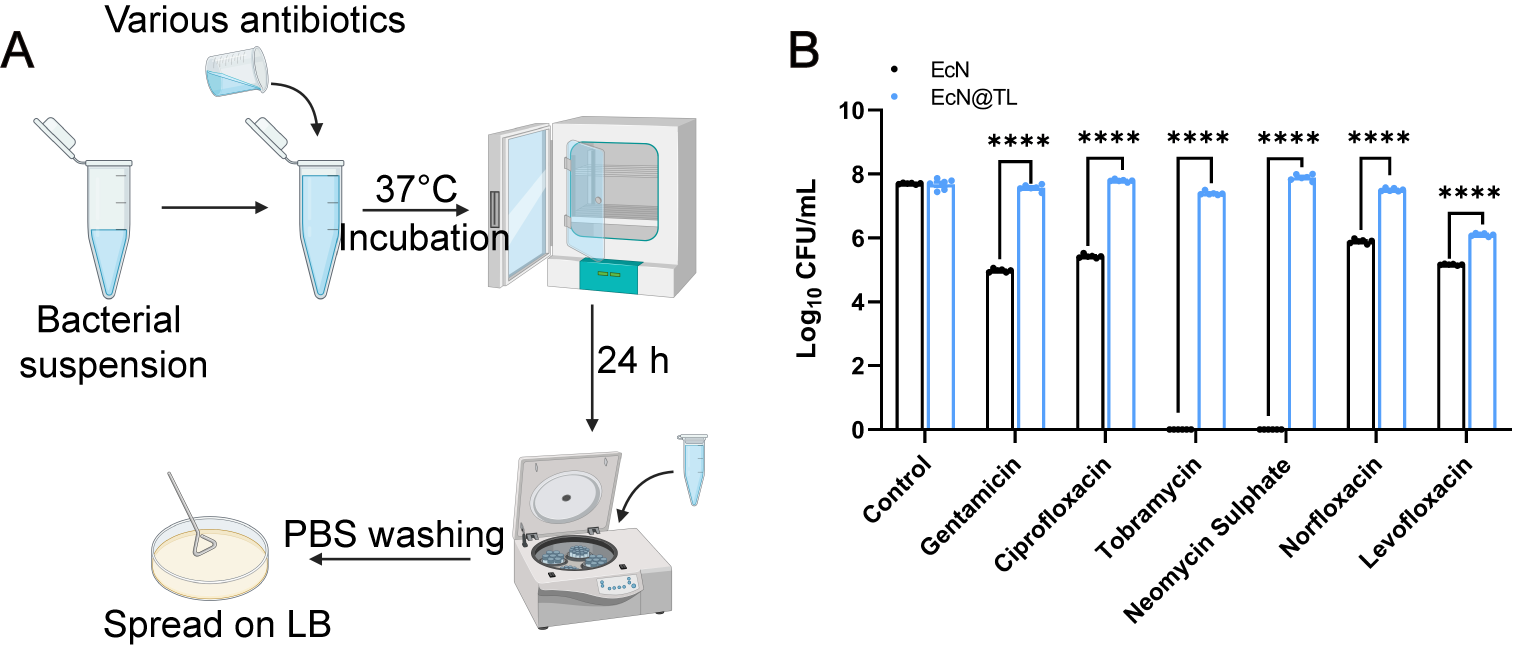


**Fig. S****14.** (A) Schematic diagram of probiotic resistance to various antibiotics. (B) Survivals of EcN and EcN@TL after the treatment with different kinds of antibiotics (n = 6). Data were presented as mean ± SEM, statistical analysis was performed using two-way ANOVA, *****P* < 0.0001.


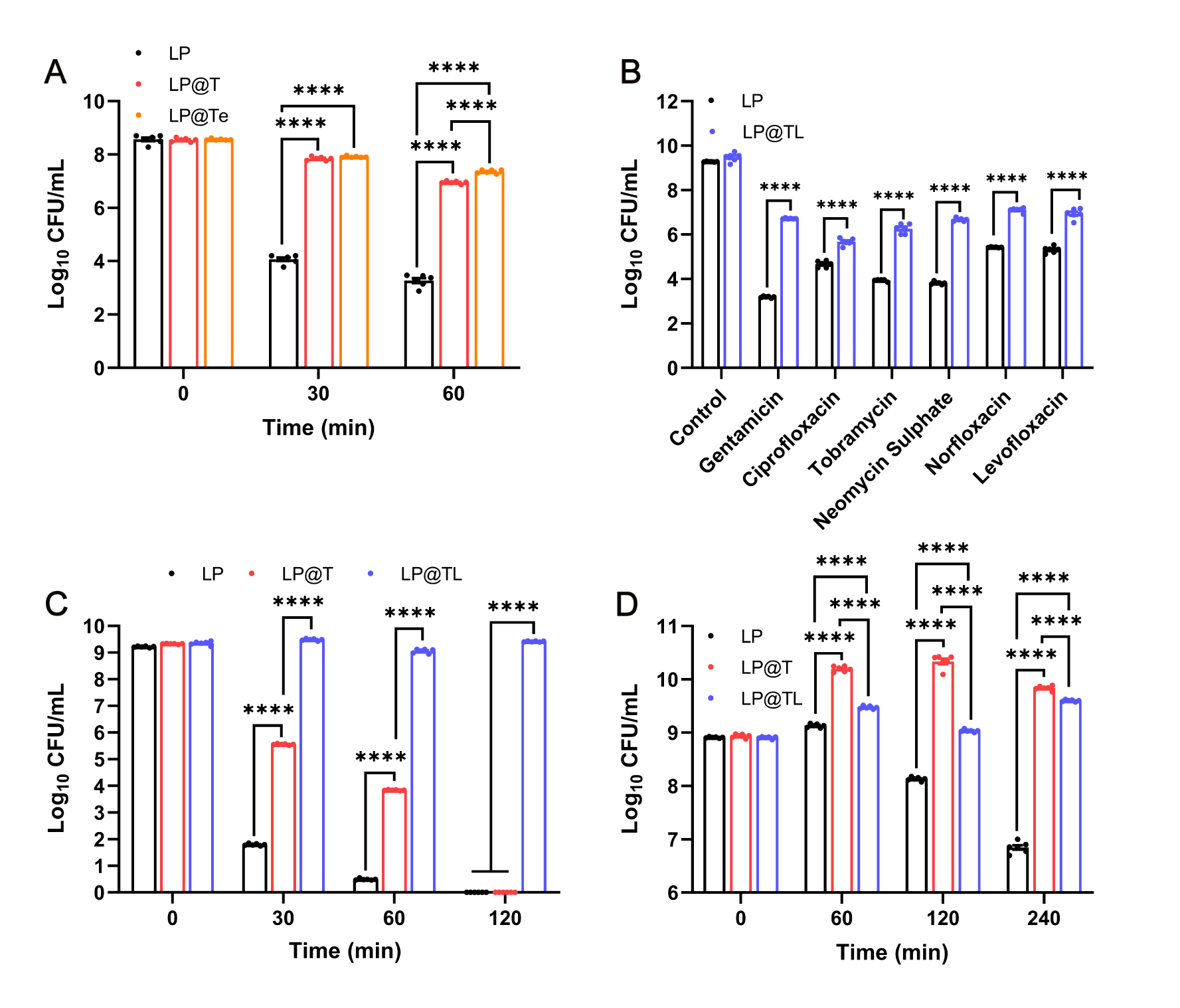


**Fig. S15.** *In vitro* resistance of LP, LP@T, LP@Te, and LP@TeL against stress environments. (A) Bacterial counts of LP, LP@T, and LP@Te after the treatment with H_2_O_2_ (15 mM, n = 6). (B) Total amount of LP and LP@TL after being treated with multifarious antibiotics (n = 6). (C) Survivals of LP, LP@T, and LP@TL after the digestion of SGF (n = 6). (D) Survivals of LP, LP@T, and LP@TL after being digested by SIF (n = 6). Data were presented as mean ± SEM, statistical analysis was performed using two-way ANOVA, *****P* < 0.0001.


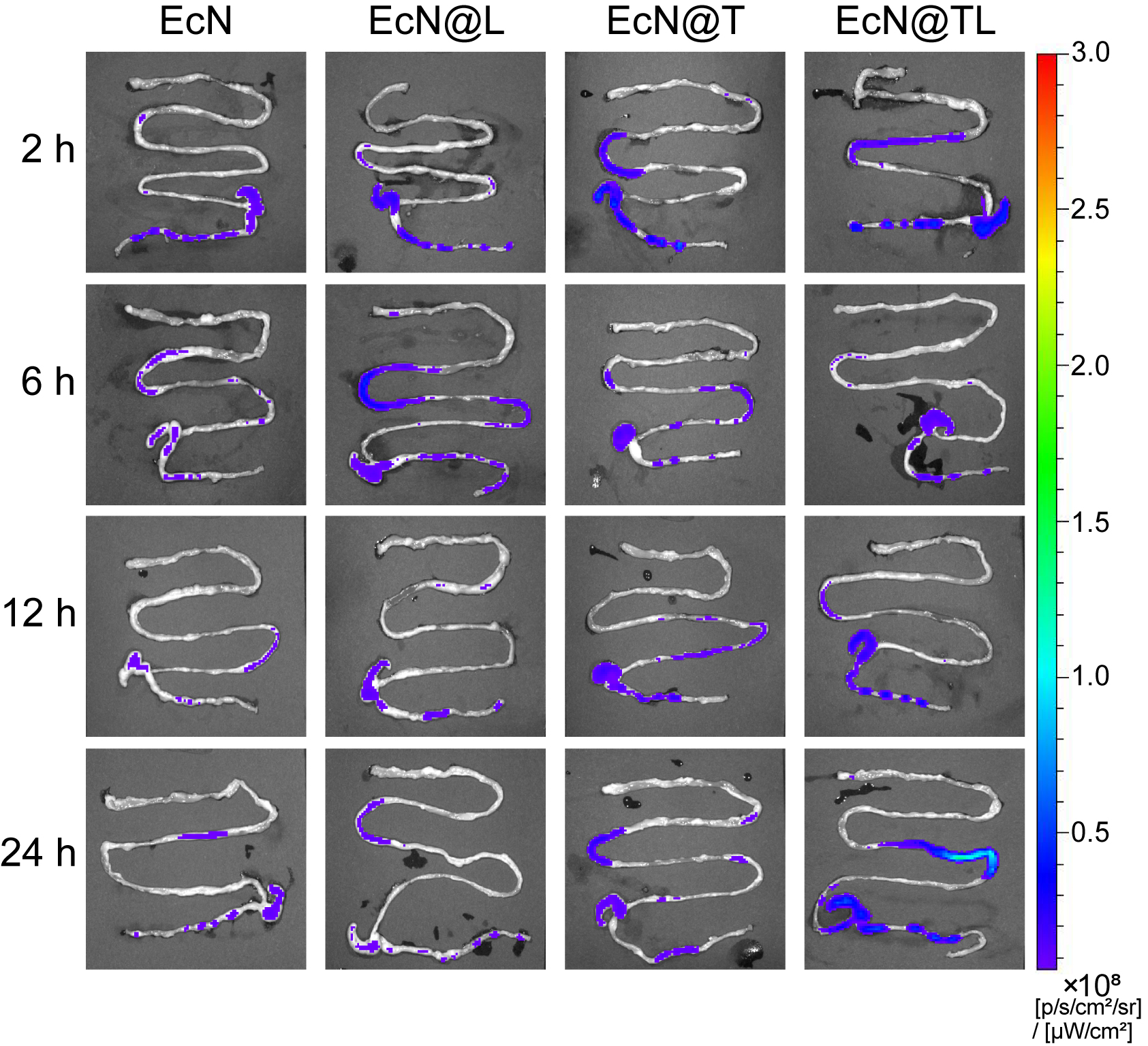


**Fig. S16.** Typical IVIS images of the whole intestine sampled at 2-24 hours after oral gavage with 5 × 10^7^ CFUs of EcN, EcN@L, EcN@T, and EcN@TL.


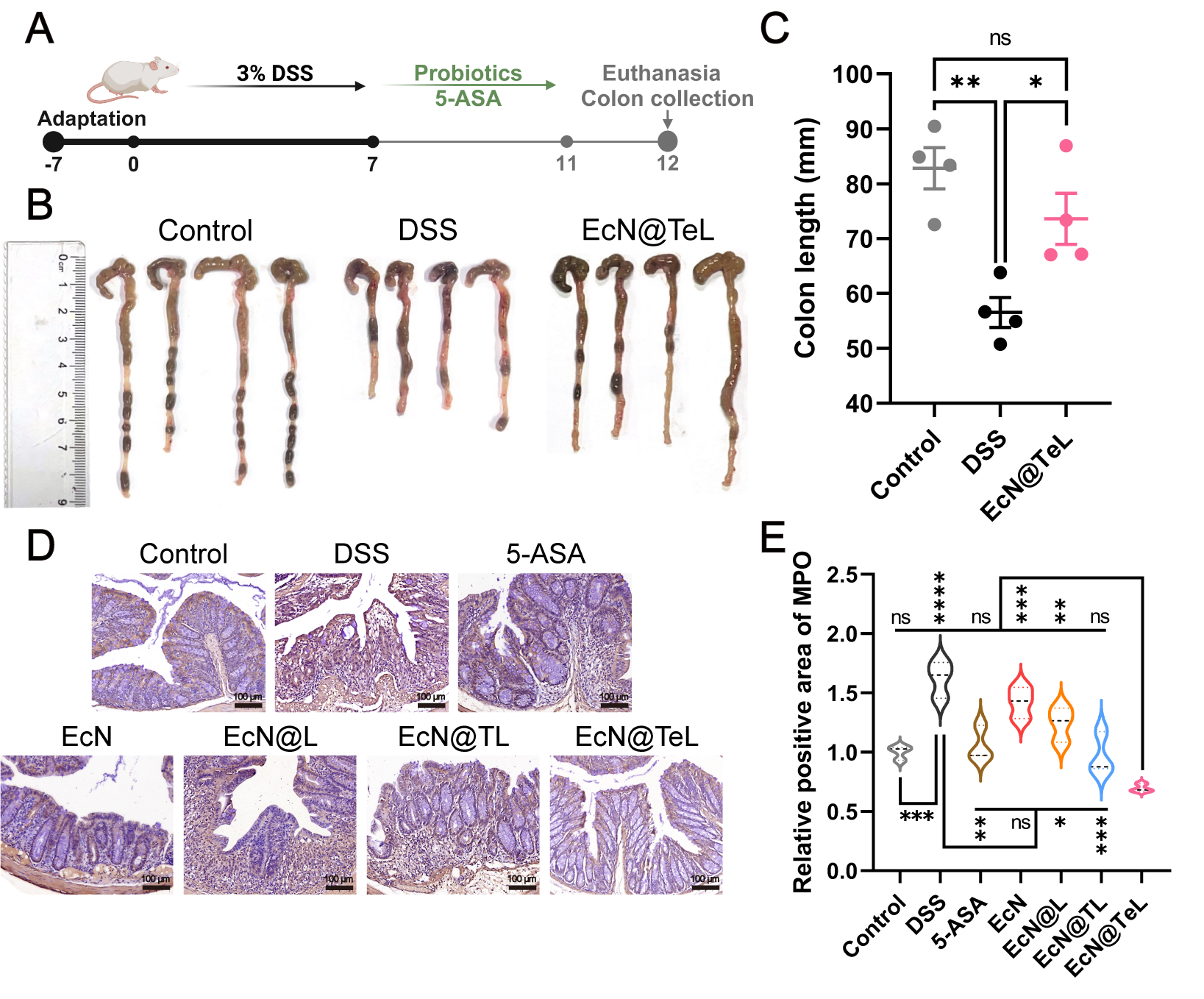


**Fig. S17.** *In vivo* palliative effect on colonic ROS of EcN@TeL. (A) Schematic diagram of experimental procedure. (B) Colon images of mice. (C) Quantitative analysis of colonic length (n = 4). (D) Typical images of colonic IHC-stained sections showed the expressive extent of MPO. Scale bars, 100 μm. (E) Quantitative analysis of the positive area of MPO (n = 3). Data were presented as mean ± SEM, statistical analysis was performed using one-way ANOVA, **P* < 0.05, ***P* < 0.01, ****P* < 0.001, *****P* < 0.0001, ns, not significant.


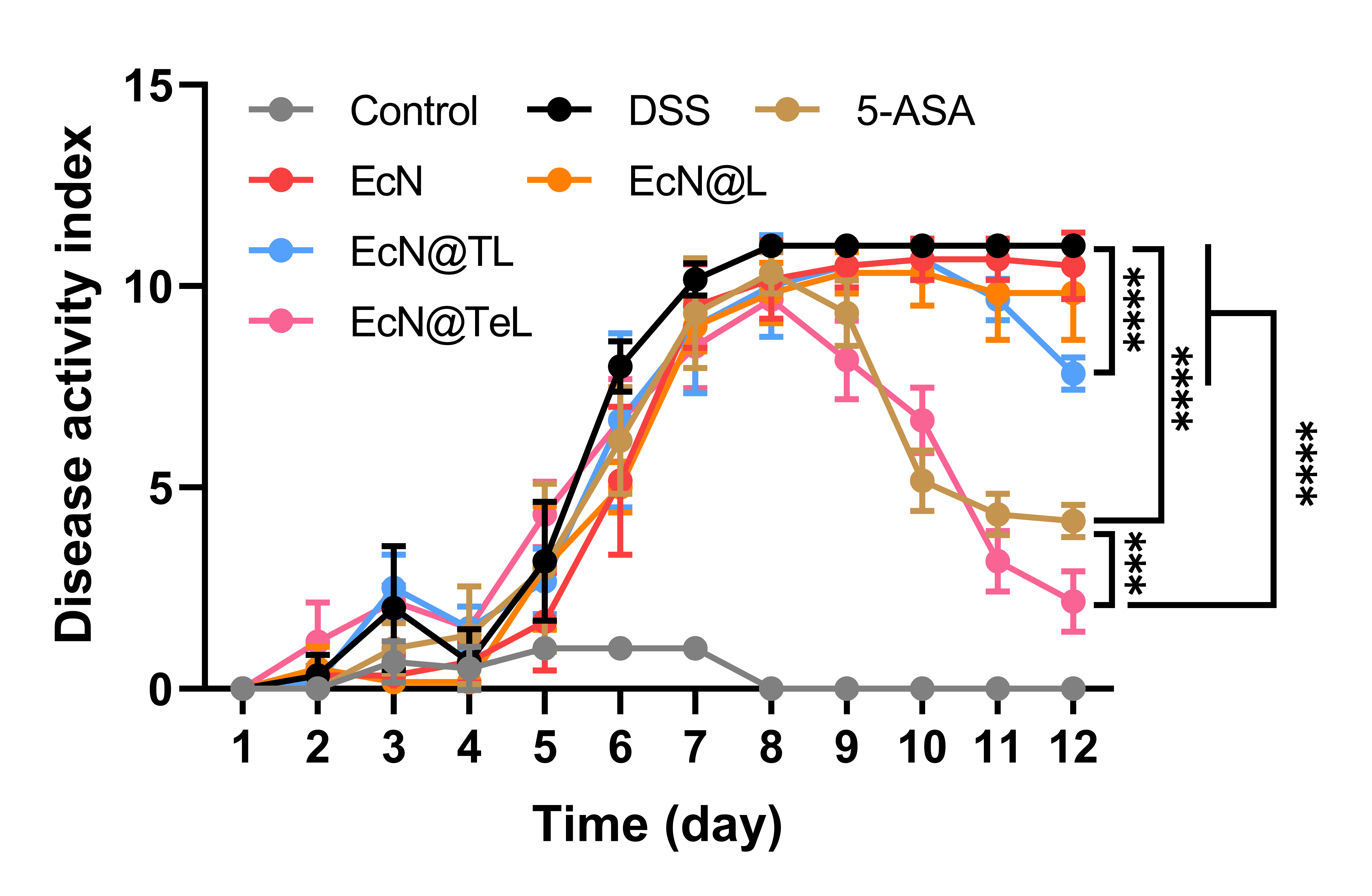


**Fig. S18.** The DAI of mice during the treatment (n = 6). Data were presented as mean ± SEM, statistical analysis was performed using two-way ANOVA, ****P* < 0.001, *****P* < 0.0001, ns, not significant.


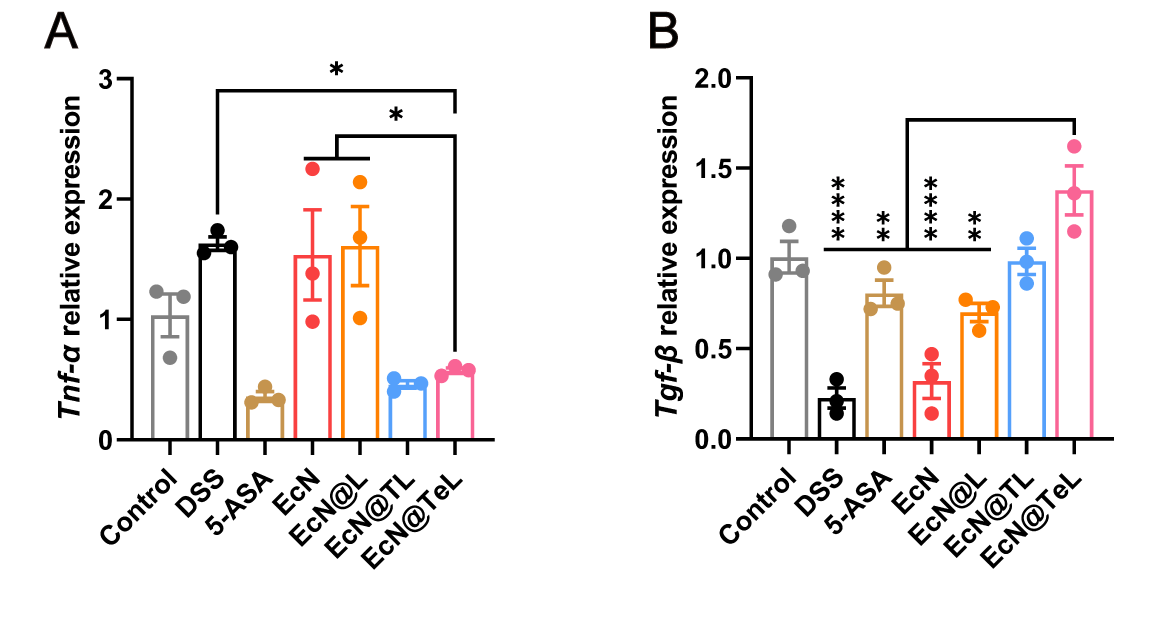


**Fig. S19.** (A) *Tnf-α* mRNA relative expression level (n = 3). (B) *Tgf-β* mRNA relative expression level (n = 3). Data were presented as mean ± SEM, statistical analysis was performed using one-way ANOVA, **P* < 0.05, ***P* < 0.01.


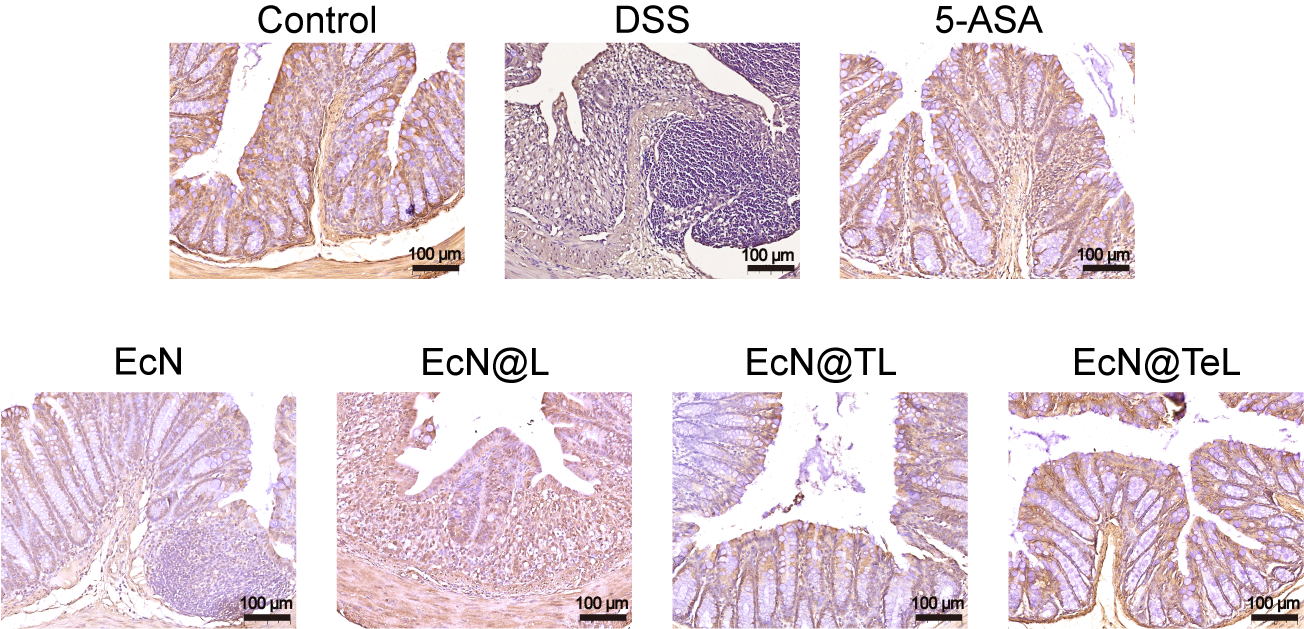


**Fig. S20.** Representative images of the immunohistochemically stained colon sections from different groups showed the expression level of ZO-1. Scale bars, 100 μm.


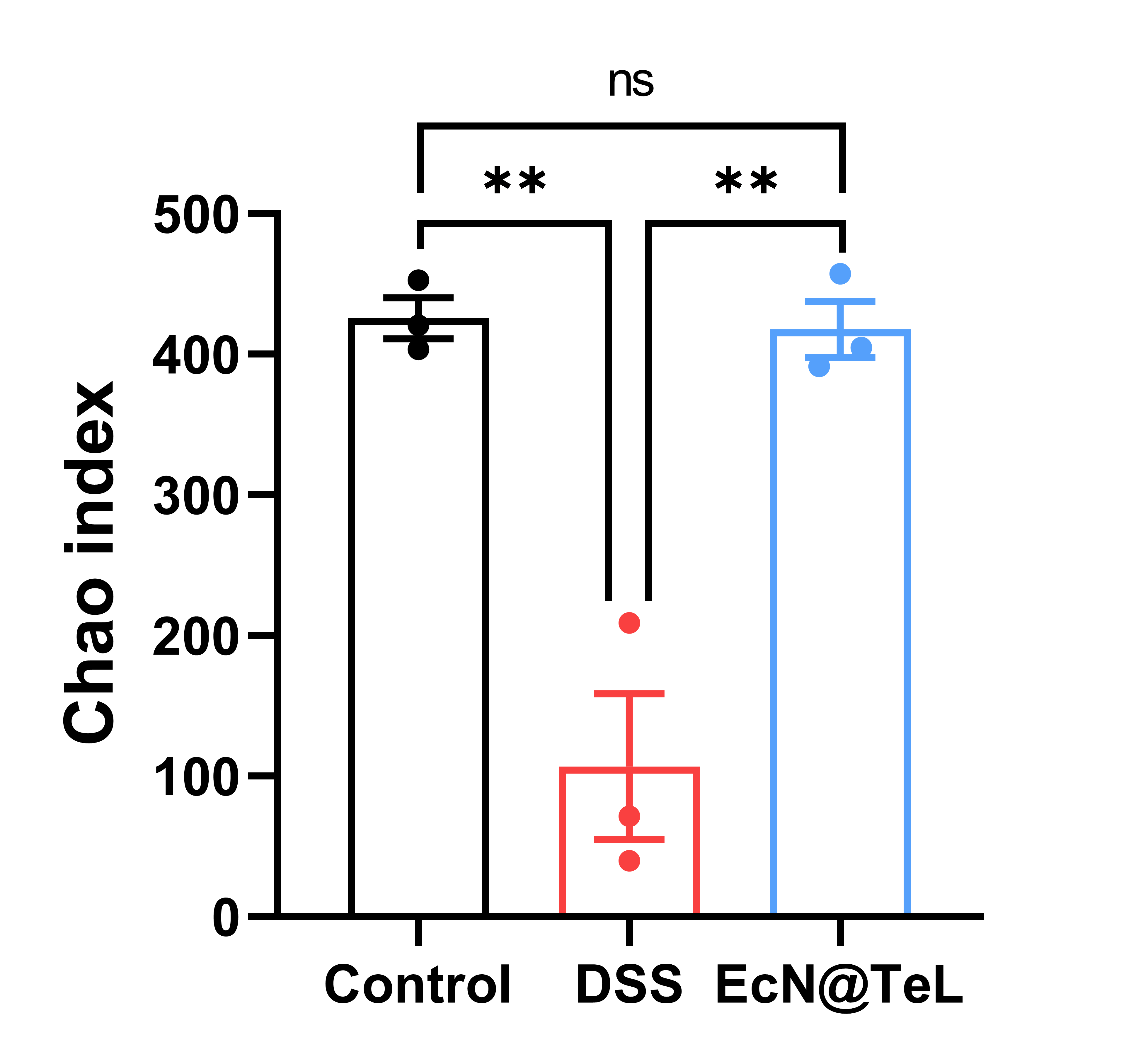


**Fig. 21.** Chao index of microbial communities with different treatments (n = 3). Data were presented as mean ± SEM, statistical analysis was performed using one-way ANOVA, ***P* < 0.01, ns, not significant.


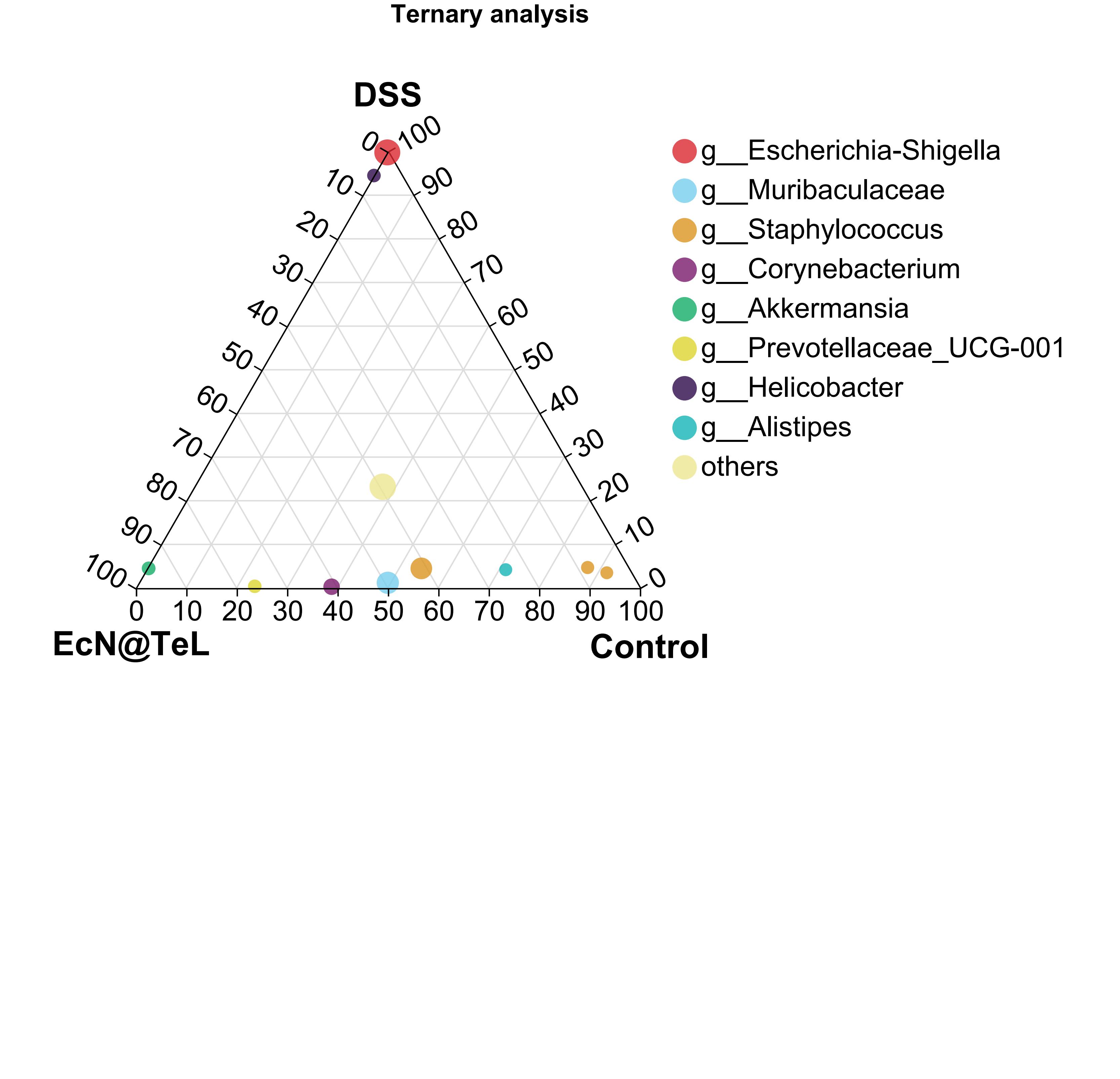


**Fig. S22.** Ternary analysis on genus level indicated the proportionate distribution of dominant species in three groups.


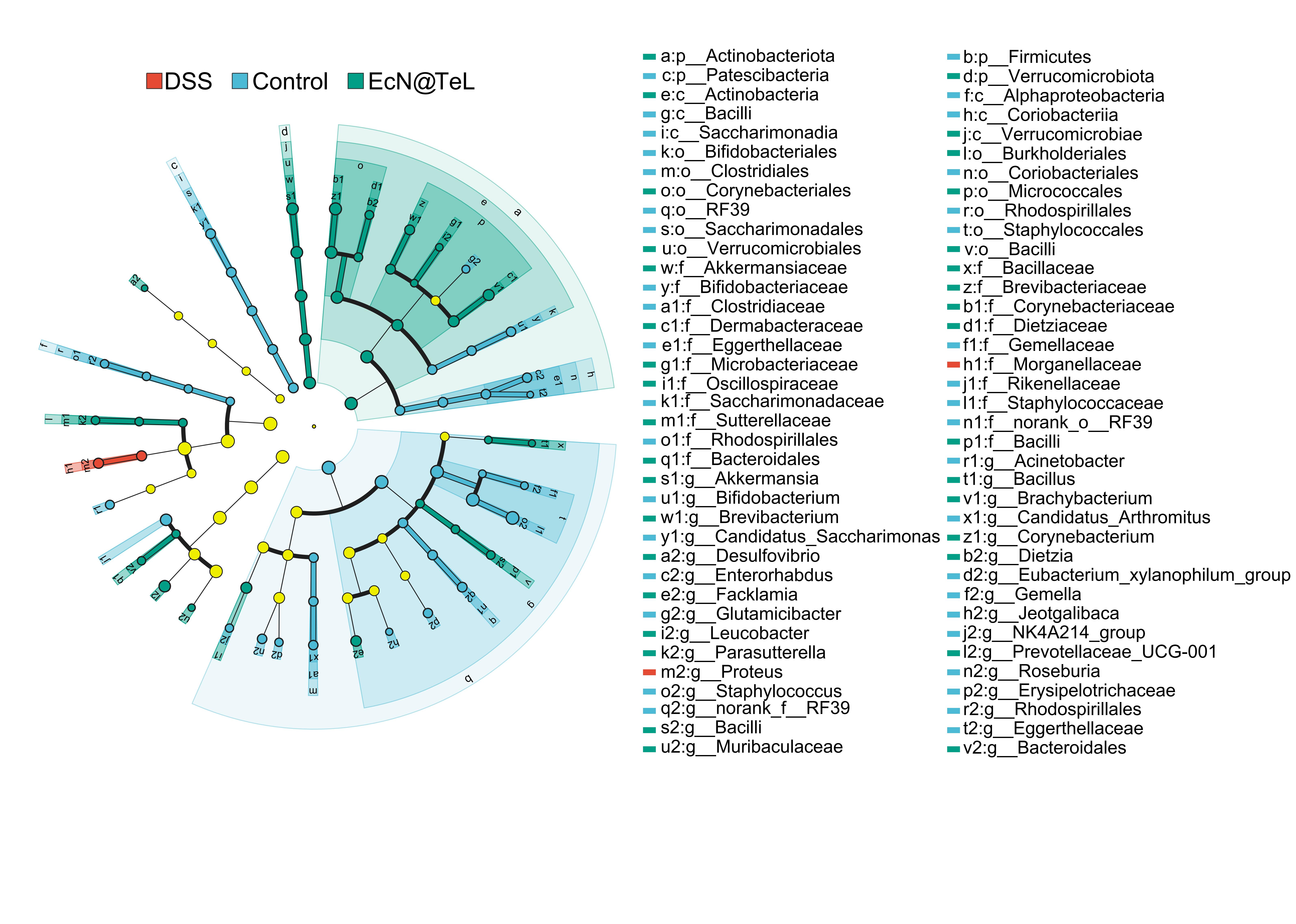


**Fig. S23.** LEfSe multilevel discriminant analysis of differences in species among three groups.


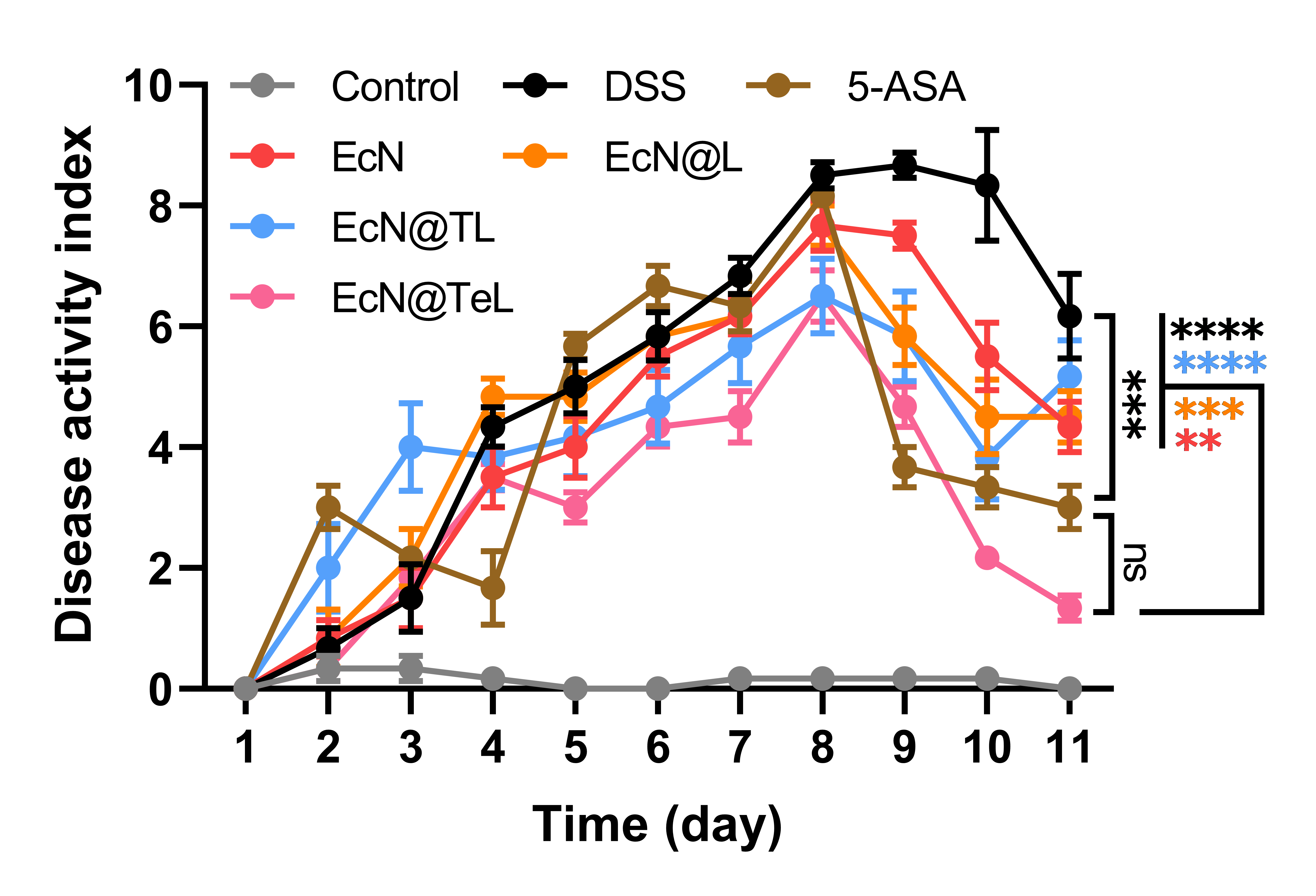


**Fig. S24.** The DAI of mice during the treatment (n = 6). Data were presented as mean ± SEM, statistical analysis was performed using two-way ANOVA, ***P* < 0.01, ****P* < 0.001, *****P* < 0.0001, ns, not significant.


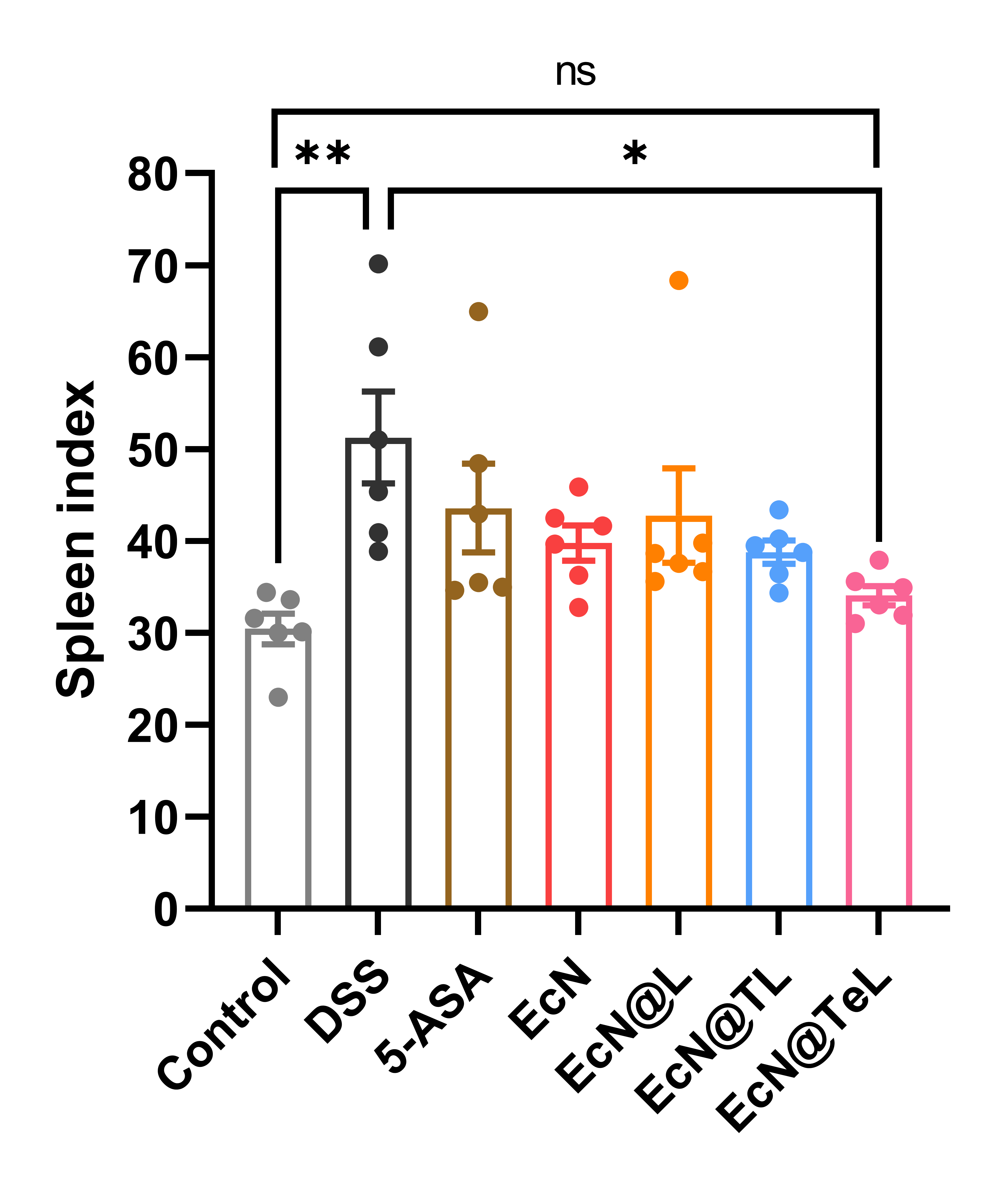


**Fig. S25.** Splenic index of mice in different treatment groups (n = 6). Data were presented as mean ± SEM, statistical analysis was performed using one-way ANOVA, **P* < 0.05, ***P* < 0.01, ns, not significant.


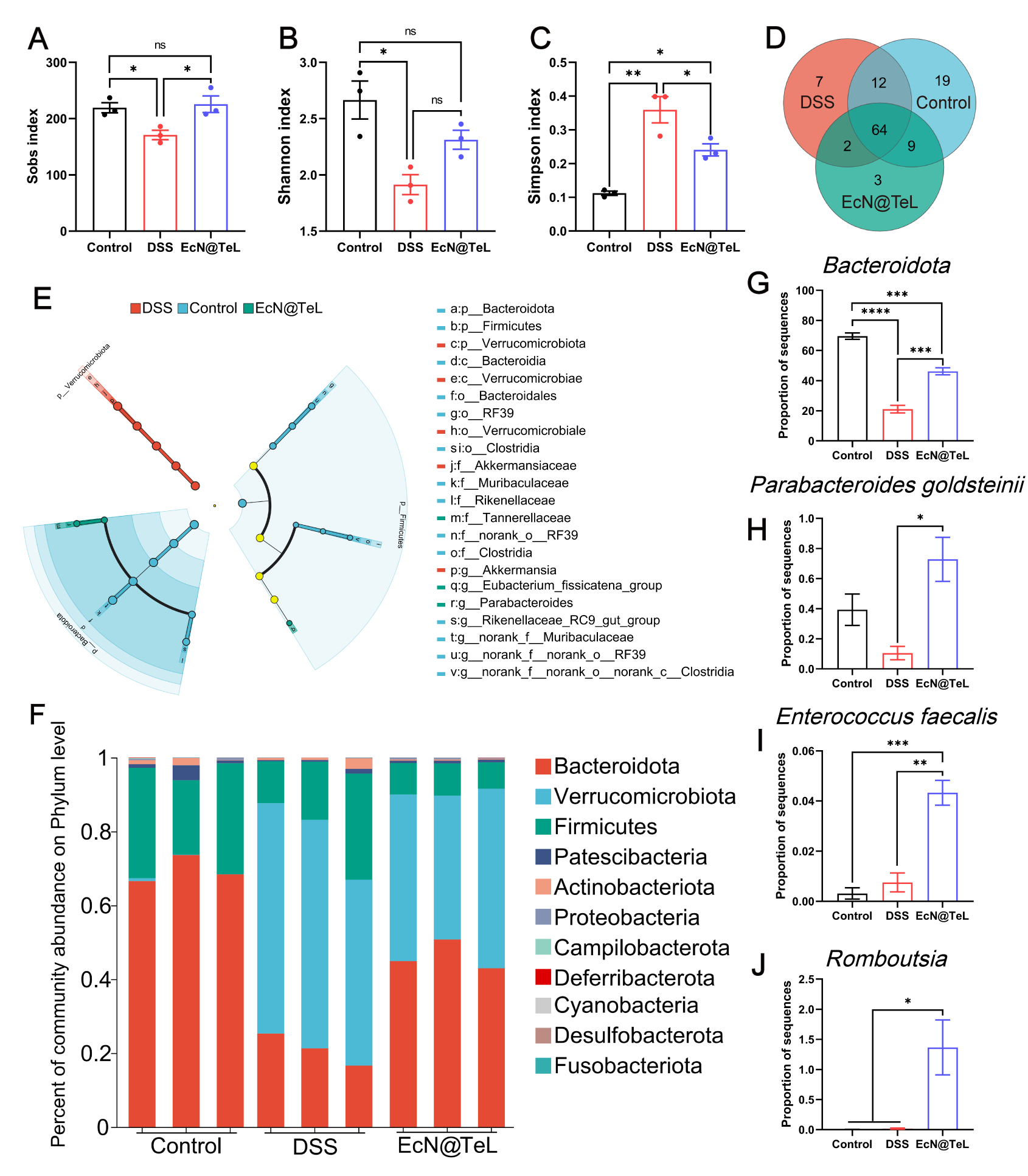


**Fig. S26.** Prophylactic regulation of EcN@TeL on intestinal flora during UC therapy. (A-C) Alpha diversity analysis of microbial communities incorporating the values of Sobs, Shannon, and Simpson (n = 3). (D) The number of species at genus level in different treatment groups. (E) LefSe multilevel discriminant analysis of differences in species among three groups. (F) Relative abundance of gut microbes at phylum level. (G-J) Relative abundance of *Bacteroidota*, *Parabacteroides goldsteinii*, *Enterococcus faecalis*, and *Romboutsia* (n = 3). Data were presented as mean ± SEM, statistical analysis was performed using one-way ANOVA, **P* < 0.05, ***P* < 0.01, ****P* < 0.001, *****P* < 0.0001, ns, not significant.

**Supplementary Tables:**

**Table S1. Disease activity index (DAI) score.**

| Score | Weight loss (%) | Fecal viscosity | Fecal Occult Blood Test |
| --- | --- | --- | --- |
| 0 | 0 | Normal | Negative |
| 1 | 1-5 | Soft stool | Wathet |
| 2 | 5-10 | Mucoid stool | Blue |
| 3 | 10-20 | Watery stool | Dark blue |
| 4 | > 20 |  | Bloody stool |

**Table S2. Histological score in the colon tissues of mice.**

| Score | Ulceration | Changes in epithelial cell | Degree of inflammatory infiltration |
| --- | --- | --- | --- |
| 0 | 0 | Normal | Normal |
| 1 | 1 | Goblet cells loss | Infiltration around crypt |
| 2 | 2 | Massive loss of goblet cells | Mucosal muscle infiltration |
| 3 | 3 | Crypt absence | Generally Mucosal muscle infiltration or incrassation |
| 4 | >3 | Severe loss of crypt or polyps regenerated | Submucosa infiltration |

**Table S3.** Primer sequence.

| Gene name | Primer sequence |
| --- | --- |
| *Actb* | F: TGTACCCAGGCATTGCTGAC  R: AACGCAGCTCAGTAACAGTCC |
| *Tnf-α* | F: TCTCATTCCTGCTTGTGGC  R: CACTTGGTGGTTTGCTACG |
| *Tgf-β* | F: GGCAGGTCTACTTTGGAG  R: GCAGGTTATCATCATCATCCC |
| *Il-1β* | F: GGCAGGTCTACTTTGGAG  R: GTAGGTCGAAGTTTAGAGC |
| *Il-10* | F: TTTCAAACAAAGGACCAG  R: GGATCATTTCCGATAAGG |
| *Muc3* | F: GCCGTGAATTGTATGAACGGA  R: CGCAGTTGACCACGTTGACTA |

*Actb*, beta-actin; *Tnf*, tumor necrosis factor; *Tgf*, transforming growth factor; *Il*, interleukin; *Muc*, mucin.
